# Supplementary material for: IDBac: an open-access web platform to identify bacteria and analyze relationships in culture collections using MALDI-TOF mass spectrometry
Source: Nat Commun. 2026 Jul 23;17:8956. doi: 10.1038/s41467-026-75579-4 (PMC13503903; doi:10.1038/s41467-026-75579-4)
Supplement: Supplementary file 2 [file 41467_2026_75579_MOESM2_ESM.pdf]

Description of Additional Supplementary Files:

Supplementary Data 1: Core identifiers for isolates uploaded to the IDBac-KB

Supplementary Data 2: IDBacKB deposition metadata template
