# Supplementary material for: IDBac: an open-access web platform to identify bacteria and analyze relationships in culture collections using MALDI-TOF mass spectrometry
Source: Nat Commun. 2026 Jul 23;17:8956. doi: 10.1038/s41467-026-75579-4 (PMC13503903; doi:10.1038/s41467-026-75579-4)
Supplement: Supplementary file 3 [file 41467_2026_75579_MOESM5_ESM.pdf]

nature portfolio

Corresponding author(s): Brian T. Murphy, Mingxun Wang, Laura Sanchez

Last updated by author(s): Jun 10, 2026

Reporting Summary

Nature Portfolio wishes to improve the reproducibility of the work that we publish. This form provides structure for consistency and transparency in reporting. For further information on Nature Portfolio policies, see our Editorial Policies and the Editorial Policy Checklist.

Statistics

For all statistical analyses, confirm that the following items are present in the figure legend, table legend, main text, or Methods section.

n/a	Confirmed	
☐	☒	The exact sample size (*n*) for each experimental group/condition, given as a discrete number and unit of measurement
☐	☒	A statement on whether measurements were taken from distinct samples or whether the same sample was measured repeatedly
☐	☒	The statistical test(s) used AND whether they are one- or two-sided<br>*Only common tests should be described solely by name; describe more complex techniques in the Methods section.*
☒	☐	A description of all covariates tested
☒	☐	A description of any assumptions or corrections, such as tests of normality and adjustment for multiple comparisons
☐	☒	A full description of the statistical parameters including central tendency (e.g. means) or other basic estimates (e.g. regression coefficient) AND variation (e.g. standard deviation) or associated estimates of uncertainty (e.g. confidence intervals)
☐	☒	For null hypothesis testing, the test statistic (e.g. *F*, *t*, *r*) with confidence intervals, effect sizes, degrees of freedom and *P* value noted<br>*Give P values as exact values whenever suitable.*
☒	☐	For Bayesian analysis, information on the choice of priors and Markov chain Monte Carlo settings
☒	☐	For hierarchical and complex designs, identification of the appropriate level for tests and full reporting of outcomes
☒	☐	Estimates of effect sizes (e.g. Cohen's *d*, Pearson's *r*), indicating how they were calculated

Our web collection on statistics for biologists contains articles on many of the points above.

Software and code

Policy information about availability of computer code

Data collection	No external software was used for data collection.
Data analysis	Data was processed by the software introduced in the manuscript. Scikit bio version 0.7.1 was used for statistical analysis. Default implementations and arguments were used for all statistical testing unless otherwise specified.

For manuscripts utilizing custom algorithms or software that are central to the research but not yet described in published literature, software must be made available to editors and reviewers. We strongly encourage code deposition in a community repository (e.g. GitHub). See the Nature Portfolio guidelines for submitting code & software for further information.

Data

Policy information about availability of data

All manuscripts must include a data availability statement. This statement should provide the following information, where applicable:

- Accession codes, unique identifiers, or web links for publicly available datasets
- A description of any restrictions on data availability
- For clinical datasets or third party data, please ensure that the statement adheres to our policy

Documentation: https://wang-bioinformatics-lab.github.io/GNPS2_Documentation/idbacdepositions/

Source Code. Source code is available in the following repositories: IDBac Analysis Interface (https://github.com/Wang-Bioinformatics-Lab/

IDBac_Interactive_Interface), IDBac Deposition Workflow (https://github.com/Wang-Bioinformatics-Lab/IDBacDeposition_Workflow), IDBac Analysis Workflow (https://github.com/Wang-Bioinformatics-Lab/IDBac_Analysis_Workflow), IDBac KB Server (https://github.com/Wang-Bioinformatics-Lab/IDBac-KB-Server). Additionally, source data are provided with this paper.

Research involving human participants, their data, or biological material

Policy information about studies with human participants or human data. See also policy information about sex, gender (identity/presentation), and sexual orientation and race, ethnicity and racism.

Reporting on sex and gender	There was no research performed on human participants in this study.
Reporting on race, ethnicity, or other socially relevant groupings	There was no research performed on human participants in this study.
Population characteristics	There was no research performed on human participants in this study.
Recruitment	There was no research performed on human participants in this study.
Ethics oversight	There was no research performed on human participants in this study.

Note that full information on the approval of the study protocol must also be provided in the manuscript.

Field-specific reporting

Please select the one below that is the best fit for your research. If you are not sure, read the appropriate sections before making your selection.

☐ Life sciences ☐ Behavioural & social sciences ☒ Ecological, evolutionary & environmental sciences

For a reference copy of the document with all sections, see nature.com/documents/nr-reporting-summary-flat.pdf

Ecological, evolutionary & environmental sciences study design

All studies must disclose on these points even when the disclosure is negative.

Study description	This study describes a crowd-sourced central knowledgebase of bacterial protein MS signatures. Accompanying the IDBac knowledgebase is analysis infrastructure to identify unknown isolates, probe relationships within culture collections, and visualize specialized metabolite differences within groups of closely related bacteria.
Research sample	The IDBac-KB consists of protein MS signatures of >1400 strains spanning 6 bacterial phyla. Samples were chosen to represent a wide taxonomic breadth to provide large coverage for a searchable database. Sample sex and age are n/a.
Sampling strategy	Samples were acquired from the strains collections of a variety of academic, government, and private institutions. These strains were not collected for this study, they already existed in collections from collaborators. Protein MS spectra of each bacterial strain were collected in biological replicates of between 3-8, which is the range of replicates found to produce the most reliable and consistent dataset. No sample size calculation was performed; this analysis is not appropriate for the current study. As we describe in the manuscript, the field is yet to achieve a "sufficient" sample size that represents a comprehensive database. To achieve this, researchers would have to document all known bacteria in the physical world. Our manuscript simply provides the infrastructure to allow this, accompanied by the beginnings of a database of bacterial protein MS spectra.
Data collection	MALDI-TOF MS data were collected on the following instruments: Autoflex Speed LRF mass spectrometer (Bruker Daltonics), rapifleX MALDI Tissuetyper mass spectrometer (Bruker Daltonics), Microflex LT MALDI-TOF mass spectrometer (Bruker Daltonics), and MALDI-8020 Benchtop mass spectrometer (Shimadzu). Nyssa Krull, Michael Strobel, Robert SHepherd, and Mandisa Timba acquired data. Instrument specifications for initial IDBac-KB populations can be found in Supplementary Table S8. Protein spectra were collected in positive linear mode and externally calibrated with Bruker Daltonics Bacterial Test Standard (BTS) or Protein Calibration Standard 1. Specialized metabolite spectra were collected in positive reflectron mode and externally calibrated with Bruker Peptide Calibration standard.
Timing and spatial scale	The majority of data in the paper were collected between January of 2024 and September of 2025, though the manuscript relies on comparing consistency between data collection from multiple time periods. Thus, the earliest data collection dates back to 12/13/2018. Statistical analyses comparing these data are listed in the SI.
Data exclusions	No data were excluded from the analyses.
Reproducibility	Protein MS spectra of each bacterial strain were collected in biological replicates of between 3-8, which is the range of replicates found to produce the most reliable and consistent dataset. Attempts to reproduce MALDI-TOF spectra of bacteria were successful.
Randomization	This category is not relevant to our study.

Blinding	In an effort to demonstrate high-throughput taxonomic annotation with batch MALDI MS acquisitions, we performed a blind analysis of 18 in-house isolates from the human gut microbiome (Fig 3, Table S1). Other analyses in the manuscript did not require blinding, as this is not applicable to NMR analyses to elucidate the structure of a natural product.

Did the study involve field work? ☒ Yes ☐ No

Field work, collection and transport

Field conditions	Relevant strain parameters are listed in detail in Supplementary Table S5. These samples were collected by the strain owner (Dr. David Carter) and any metadata associated with collection are deposited in the publicly accessible IDBac-Knowledgebase.
Location	Samples were collected from Chaminade University of Honolulu. Relevant strain parameters are listed in manuscript methods section and in detail in Supplementary Table S5.
Access & import/export	Samples used in Figure 4 analysis were obtained from the laboratory of Dr. David Carter, while at the Chaminade University of Honolulu. All carcasses were acquired commercially (Island Farms, Waianae, USA) and handled in accordance with State of Hawaii Statute §159–21. For all other bacterial isolates described, no collection was carried out.
Disturbance	No disturbances were caused in the carrying out of this study.

Reporting for specific materials, systems and methods

We require information from authors about some types of materials, experimental systems and methods used in many studies. Here, indicate whether each material, system or method listed is relevant to your study. If you are not sure if a list item applies to your research, read the appropriate section before selecting a response.

Materials & experimental systems

n/a	Involved in the study	
☒	☐	Antibodies
☒	☐	Eukaryotic cell lines
☒	☐	Palaeontology and archaeology
☒	☐	Animals and other organisms
☒	☐	Clinical data
☒	☐	Dual use research of concern
☒	☐	Plants

Methods

n/a	Involved in the study	
☒	☐	ChIP-seq
☒	☐	Flow cytometry
☒	☐	MRI-based neuroimaging

Plants

Seed stocks	No plant materials were used in this study.
Novel plant genotypes	No plant materials were used in this study.
Authentication	No plant materials were used in this study.
