# Supplementary material for: IDBac: an open-access web platform to identify bacteria and analyze relationships in culture collections using MALDI-TOF mass spectrometry
Source: Nat Commun. 2026 Jul 23;17:8956. doi: 10.1038/s41467-026-75579-4 (PMC13503903; doi:10.1038/s41467-026-75579-4)
Supplement: Supplementary file 4 [file 41467_2026_75579_MOESM6_ESM.pdf]

nature portfolio

Peer Review File

IDBac: an open-access web platform to identify bacteria and analyze relationships in culture collections using MALDI-TOF mass spectrometry.

Corresponding Author: Dr Brian Murphy

This file contains all reviewer reports in order by version, followed by all author rebuttals in order by version.

Version 0:

Reviewer comments:

Reviewer #1

(Remarks to the Author)
This manuscript presents IDBac, a web-based platform that integrates MALDI-TOF mass spectrometry data—both protein "fingerprints" and specialized metabolite profiles—into an open-access spectral library for bacterial identification and comparative phenotyping. The authors aim to extend MALDI-TOF applications beyond clinical microbiology into environmental and natural product discovery contexts. The concept of bridging microbial identification with metabolomic potential is both ambitious and valuable, and the effort to construct a public, community-driven repository deserves recognition. If implemented rigorously, such a platform could substantially influence how microbiologists dereplicate isolates and assess chemical novelty. While the idea and motivation are commendable, several critical weaknesses prevent the study from realizing its potential. The manuscript currently presents a technically interesting but underdeveloped platform description rather than a mature, validated system with clear biological or methodological contributions.

1. Library Strain Diversity and Metadata Limitations
The strength of any identification platform lies in the depth, breadth, and curation quality of its reference library. Although the authors have assembled a dataset of approximately 1,400 bacterial isolates, the library appears highly uneven in both taxonomic representation and environmental provenance. Based on Table 1 and the Supplementary Dataset, the majority of entries originate from a narrow range of institutional sources, including the Duchossois Family Institute, NRRL, and a few collaborating academic laboratories. As a result, the dataset is heavily weighted toward a limited set of genera, notably Bacillus and Streptomyces, as well as other Actinobacteria/Firmicutes commonly associated with natural product research.

This bias is problematic for several reasons. First, it undermines the utility of IDBac as a general-purpose identification tool: users attempting to classify isolates from underrepresented phyla (for example, Proteobacteria, Cyanobacteria, or Bacteroidetes) will likely encounter reduced accuracy or no meaningful match at all. Second, without representation from diverse ecological and geographical origins, the system's capacity to reveal relationships between microbial habitats and metabolic signatures remains constrained. In short, the current dataset provides a promising proof of concept, but not yet a comprehensive or balanced identification compendium.
Equally concerning is the lack of standardized and complete metadata. Many entries lack detailed contextual information, including source habitat (e.g., soil, marine, host-associated), precise isolation site, and sampling conditions. This omission limits not only the interpretability of spectral clustering but also the scientific reproducibility and downstream utility of the database. Provenance metadata are fundamental for correlating phenotypic variation with ecological or evolutionary patterns. In its current form, the IDBac repository provides little indication that metadata completeness was prioritized or systematically curated.

2. Narrative Focus and the Specialized Metabolite Discovery Section
While integrating protein and specialized metabolite profiles under one platform is conceptually attractive, the metabolite discovery vignette currently distracts from the manuscript's core purpose. The primary contribution of IDBac should be its capacity to facilitate high-throughput microbial identification and comparative analysis; however, the inclusion of a "new metabolite discovery" story shifts attention toward an isolated chemical finding that lacks sufficient experimental substantiation.

According to the manuscript, a distinct m/z feature was detected in a subset of strains and tentatively proposed as a novel metabolite. Yet no structural confirmation, MS/MS analysis, or bioactivity validation accompanies this claim. Without such evidence, the observation remains preliminary—essentially a pattern recognition result. Presenting it as a discovery risks overinterpretation and blurs the distinction between what IDBac can suggest versus what it can demonstrate.

Moreover, devoting an entire section to this anecdote unbalances the manuscript's narrative flow. Readers expecting a methods-centered presentation are suddenly confronted with a tangential storyline that feels incomplete. The result is a paper that tries to tell two stories—database construction and metabolite discovery—without fully delivering on either.

3. Technical Limitations of MALDI-TOF for Small Molecule Identification
The manuscript exhibits an overly optimistic interpretation of MALDI-TOF's capabilities for small-molecule analysis. While the platform's novelty lies in extending MALDI-TOF beyond the protein fingerprinting range, the technical constraints inherent to the method must be acknowledged with candor.

Resolution and mass accuracy limitations. Standard benchtop MALDI-TOF instruments (e.g., Bruker Microflex, Shimadzu Axima) provide modest mass resolution (500–1000 at m/z 500) and limited mass accuracy. This inherently restricts their ability to distinguish closely spaced molecular ions or isotopic variants. Without high-resolution capability (such as MALDI-TOF/TOF or MALDI-FTICR), any identification based solely on nominal m/z is speculative.

Matrix interference. The use of CHCA or similar matrices introduces dense background signals below m/z 700, masking many small metabolites. Even above this range, adduct formation and variable ionization efficiencies produce inconsistent spectra. Since the study employs a single matrix for both protein and metabolite detection, matrix bias could be substantial. Yet this factor is scarcely discussed in the manuscript.
Lack of fragmentation data. MALDI-TOF profiling, without tandem MS, cannot provide structural confirmation. Thus, any identification or dereplication claim derived purely from intact mass data should be considered putative.

4. Standardization Across Instruments and Reproducibility
A fundamental challenge for any shared spectral database lies in ensuring cross-instrument comparability and reproducibility. The manuscript currently lacks sufficient evidence to demonstrate that IDBac can deliver consistent results across different MALDI-TOF instruments, laboratories, or operators. Without such validation, claims of general utility remain speculative.

Data harmonization is particularly critical because MALDI spectra are sensitive to numerous variables: laser energy, matrix crystallization, sample spot uniformity, and calibration accuracy. Even identical instruments can yield subtly different peak patterns, depending on the user's technique or maintenance state. Moreover, the manuscript indicates that data were collected using instruments from multiple manufacturers. While the authors note that spectra were converted to mzML and processed uniformly, there is no quantitative assessment of the effectiveness of these harmonization steps.

Equally concerning is the absence of a formal reproducibility analysis. The authors do not report whether replicate spectra of the same strain (either technical or biological) consistently cluster together. Without such metrics, it is challenging to assess the platform's reliability for identification purposes.

5. Statistical Rigor and Evidentiary Strength
The interpretation of Figure 4 exemplifies a broader issue with statistical and evidentiary rigor throughout the manuscript. The figure—apparently a clustering or comparative visualization—illustrates intriguing patterns, yet the conclusions drawn from it are largely qualitative and lack statistical validation.

Suppose the authors intend to show that IDBac spectral clustering reflects taxonomic or functional groupings. In that case, the analysis must be supported by metrics such as PERMANOVA, ANOSIM, or adjusted Rand indices comparing MALDI-based clusters to 16S phylogeny or known taxonomy. Without such quantification, the apparent separation in Figure 4 could reflect arbitrary scaling or sample composition differences rather than true discriminatory power.

Similarly, if Figure 4 highlights differences in metabolite profiles, these observations must be supported by replicate consistency and statistical tests. The manuscript currently lacks any indication that such tests were performed.

(Remarks on code availability)

Reviewer #2

(Remarks to the Author)
I read the manuscript entiteled "IDBac: an open-access web platform and compendium for the identification of bacteria by MALDI2 TOF mass spectrometry" with great interest and believe that its discussion of a highly timely and relevant subject will appeal to a wide readership. In my view, the manuscript is well written and logically structured. The authors present IDBac, an open access platform which can be used to identify bacteria based on MALDI-ToF mass spectrometry. In addition to this, IDBac represents an analysis infrastructure that offers advanced, non-standard possibilities for MALDI-ToF mass spectrum analysis that enables also the visualisation of specialized metabolite differences.

Unfortunately, based on the materials provided, I am currently unable to provide a comprehensive assessment of the manuscript. Despite my efforts to register on the IDBac web platform, I have not yet been granted access to the system. I fully understand the authors' decision to restrict the use of IDBac to registered users only, even though comparable online platforms such as MicrobeNet handle this differently. However, either the registration process for IDBac should be simplified, or reviewers should be given trial access to the IDBac platform from the outset.

Without access to IDBac, I am unfortunately unable to provide a qualified assessment of the manuscript. I would therefore be grateful if you could send me the access information for the IDBac web resource. I am really interested in testing IDBac, so once I have received the credentials, I will provide the evaluation of the submitted manuscript promptly.

(Remarks on code availability)
The provided link to Github did not work - page not found - 404 error? Authors, please check the link.

https://github.com/Wang-Bioinformatics-Lab/IDBacDeposition_Workflow

Reviewer #3

(Remarks to the Author)
The manuscript describes the web platform rollout of IDBac, a tool that has existed for some time now, and this interface significantly increases the usability and user experience. I think the authors have provided sufficient novelty from previous manuscript detailing IDBac and the online interface makes this tool more accessible and FAIR. Like much of the work in natural products research, borrowing on the medical community advances, IDBac seems to provide users with the ability to rapidly ID strains, nullifying some of the research expense that comes with sequencing. One could argue that the necessity for the instrumentation is equally a limitation for those researchers outside of North America, Europe, and Asia.

Two questions regarding the tool and workflow, (1) is metadata curation done under controlled vocabulary, to enable more efficient mining of the repository. This is an essential step in ensuring interoperability across the database, making future use by you as the creators and the users much more efficient for providing statistics and re-analysis. (2) While many MS-based tools have little QC, something like this that could be used to ID strains maybe should have some level of QC, especially if users may use it in context with pathogenic strains where IDs may shift perception of required biosafety levels. Is there a precedence from clinical biotyping to standardizing and determine data quality, especially across labs? Or is this something that could be developed in the future (addition of specific inexpensive standards that are then QC checked by the IDBac system, without which data is rejected?) I don't think these hold back the current advancement but enhance the analysis and should be considered for the future.

I believe the analyses have covered a wide breadth of applications and show the power of utilizing such a workflow, especially showing the timescale study in Figure 4C. Do you have % variability explained for this plot?

For the MANs, I think this is a valuable asset to now have integrated into the IDBac system. It builds from the vast knowledge of chemotaxonomy that has existed for some decades in plants and fungi and I think you need to add some of this context to the manuscript. There are multiple reviews that exist and I will let the authors decide what is most appropriate. I also believe the MANs are slightly reflective of a preprint describing metadata-based molecular networks (https://chemrxiv.org/engage/chemrxiv/article-details/6687b32d5101a2ffa875e356) and I think you have to acknowledge the existence of this development, as it mirrors mapping features on selected metadata groups (in your case phylogenetic metadata).

The section from lines 298-309 is effective but more a reflection of the poor quality of 16S to distinguish species (which is widely acknowledged for genera with many species) than the distinguishing power of metabolomes to differentiate species, again leaning on chemotaxonomy as an entire field. I believe this could be reframed in an explicit way, 16S fails us and WGS is expensive on larger scales, therefore using metabolomes to differentiate species is an efficient means similarly to proteomics. Does your proteomic data reveal a difference between these two species as well or do you only get this taxonomic differentiation with the metabolomics? I believe in regards to this assessment, the subheading title needs to be changed to species discrimination, as the two Paraburkholderia were never the same species in the first place.

In figure 5A, highlight where in the MAN compounds 1 and 2 are, this reinforces the technology.

Structure elucidation is well done, you should deposit the NMR data into NP-MRD (https://np-mrd.org/) if not done so already. NMR data in supplementary essential as screenshots are not very useful and this repository makes it more accessible.

(Remarks on code availability)
I am not an expert in coding and therefore cannot elaborate on its quality.

Version 1:

Reviewer comments:

Reviewer #1

(Remarks to the Author)
The manuscript has been revised extensively and has improved considerably. The authors' point-by-point responses are appropriate and resolve the issues raised. I have no further comments on the revised manuscript.

(Remarks on code availability)

Reviewer #2

(Remarks to the Author)
NCOMMS-25-72074-T, " IDBac: an open-access web platform and compendium for the identification of bacteria by MALDI2 TOF mass spectrometry" by Krull et al.

In this work, Krull and his colleagues introduce a web-based platform, IDBac, which is described to allow not only identification of bacteria based on their MALDI-ToF mass pattern, but also probing taxonomic relationships between them, and visualizing MALDI differences of secondary metabolite signatures produced by closely related bacteria. To this end, the authors have created a reasonably comprehensive MALDI-ToF spectrum database and present in their manuscript various tools for spectrum analysis and visualization. This ambitious undertaking is intended to be expanded in the future with data from external users and represents a potentially interesting extension of analysis options for more than just MALDI-ToF-MS identification of bacteria.
I very much welcome the idea and motivation behind the project, but I must note that the manuscript does not appear to be sufficiently focused in its current form. It is my general impression that the authors are more interested in including and presenting a high number of functions and methods for analyzing bacterial MALDI spectrum data rather than on usability and simplicity.
This not only detracts from the clarity and comprehensibility of the manuscript. Additionally, the IDBac (or better the GNPS2) web platform appeared to me somewhat confusing. In my opinion, users without an extensive IT background will only be able to use it after a lengthy training period. Both the manuscript and the IDBac platform could benefit from focusing on a few, selected core functions.
My comments and criticisms are detailed below.
After the authors provided me with the login details for the IDBac platform in response to my request in the first review, I was able to log into the platform as idbac_reviewer2 using the credentials supplied. This worked quite well, but unfortunately my testing were unsuccessful. My original intention was to challenge the IDBac spectrum library by own spectrum data (BrukerRaw format) from selected Paraburkholderia species from which I knew they are represented in the IDBac database - after all, the authors had raised my expectations to do so by the very first sentence of their abstract: "The identification of bacteria is central to the microbiological sciences"
I do not want to go into all the details of my test here, but I found that the IDBac-Analysis workflow is just one out of 70 workflows hosted on the GNPS2 Analysis Hub website. Furthermore, I learned that potential IDBac users must first convert their spectrum data according to what seemed to me to be a rather complex scheme. I might have achieved a positive result if I had invested more time in testing IDBac and/or used the IDBac support. However, compared to MicrobeNet, a similar solution for the taxonomic analysis of MALDI-ToF mass spectra that I use regularly, I found that IDBac has some weaknesses in terms of efforts, user-friendliness, and usability. Although the manuscript points out several times that IDBac has a significantly larger range of functions than alternative solutions, probably MicrobeNet is meant, it left me with the impression that using IDBac requires in-depth prior knowledge. The GNPS2 Analysis Hub indeed offers many different, potentially useful functions. However, as a user seeking to identify bacteria based on their MALDI pattern, I found the website rather confusing and did not always understand the purpose of the workflows hosted.
Unfortunately, my attempts to perform analyses using sample files stored on the website (spectrum data from the authors or their cooperation partners) were unsuccessful as well. Since no error messages were displayed, I stopped trying to use the IDBac website after about 90 minutes. However, I will not blame the authors for this failure in this review. It is possible that I overlooked or did not understand all relevant aspects. Nevertheless, the authors should bear in mind that their offering is essentially aimed at microbiologists who want quick results with minimal time and effort. In my opinion, IDBac's success will depend mainly on the user experience when visiting the website for the first time, not on the range of functions offered.

Major Comments
1. Abstract, lines 60-61. The phrase "… would facilitate strain identification" should be replaced by " … would facilitate identification at the species and in some instances at the subspecies level".
I do not support the opinion that MALDI-ToF MS is a tool that generally allows strain identification. In my experience, contrary to a number of over-optimistic studies, MALDI-ToF MS only shows limited taxonomic resolution. MALDI-ToF MS has a taxonomic resolution that is limited to the species level, unlike methods such as WGS, FTIR, or Raman spectroscopy. Sometimes, it cannot even differentiate between related microbial species. For example, MALDI-ToF MS cannot reliably distinguish between selected B. cereus group species, E. coli and Shigella, or Citrobacter freundii complex species. To avoid misunderstandings, it should be noted that the latter statement does not mean that MALDI-ToF MS can never distinguish between different strains, biovars, or subspecies within a species.

2. The results on line 159 state that "IDBac represents the largest and most comprehensive open-access MALDI-TOF protein knowledge base to date." This statement does not correspond with the facts. The MicrobeNet database, for example, is also openly accessible and can be used without registration, though they require Bruker-formatted XML data files for testing. For instance, MicrobeNet's CDC databases (CDC, CDC Tick, and CDC Unidentified Bacteria) contain a total of 2,240 MSPs: 1,647, 501, and 92, respectively. Second, the RKI database of highly pathogenic bacteria, which is freely available on ZENODO (Lasch et al., 2025), has 1,599 strain entries, exceeding IDBac's 1,418. The EMALiMB database (Ban et al., 2025; RIKEN Institute, Japan) contains 1,736 MSPs. The MALDI-UP spectrum catalog, a user spectrum exchange platform based in Stuttgart, Germany, lists 8,565 entries (version 2026-01-13) from a variety of different taxa. Finally, MALDI-ToF data collections such as MARISMA (Schmidt-Santiago et al., 2024) and DRIAMS (Weiss et al., 2022) contain gigabytes of well-characterized bacterial mass spectra of clinically relevant pathogens for AMR profiling (DRIAMS contains 145 GB of raw spectrum data!).
Apart from these figures, it should also be noted that the IDBac database is dominated by strains and spectra mainly from Actinomycota. For instance, Streptomyces, Microspora, and Bacillus account for over half of all IDBac entries (see Figure 2B). (Figure 2B).
Therefore, please remove the misleading statement in line 159 (and all other instances) from the manuscript.

3. Cosine distance (on a 0-1 scale, 0 signifies identical). To me it is not clear how exactly the calculations are performed and why the cosine distance and not some other distance measure is preferred. What exactly are the advantages of this type of distance over other distances like Euclidean, Hamming, Manhattan, correlation-based distances, etc.? I also consider it a disadvantage when a universal threshold cannot be defined (cf. line 192: “there is no universal threshold to define genus/species boundaries between taxa”), as is the case with the significantly more complex score values in Bruker's MALDI Biotyper (MBT). The MBT defines such general thresholds, and the identification results are color-coded according to a traffic light scheme. This has major advantages for end users, but admittedly does not always work consistently in the MBT either. When analyzing the dendrogram of Figure 3 it appears to me that cosine distances are perhaps not optimally suited for discrimination of different bacterial taxa. The dendrogram given suggests that some species are discriminated at distances ~0.80 (e.g. some Bacterioides) while phylogenetically much more different taxa like Mediterraneibacter (Class: Clostridia) and Lactococcus (Class: Bacilli) are separated by a similar cosine distance of 0.81. Therefore, I do not consider this characteristic of the dendrogram to be particularly advantageous. However, these variable thresholds do not necessarily have to be based on the distance metric used; rather, they could be attributed to the type of preprocessing applied or the cluster analysis method used. So, please explain exactly how the data was preprocessed for hierarchical clustering. Similar to what is described starting at line 505? In this context, it is important to know if peak tables were compared as assumed (as it is done with the MBT/Bruker). If so, how many peaks were selected from each spectrum? Was it ensured that the number of peaks per spectrum corresponded to a specified number?

4. The complete list of strains should be made available at the IDBac / GNPS2 Analysis Hub website.

5. Dendrogram of Figure 3. Unfortunately, the strain names are missing, so it was unclear to me if the different Mediterraneibacter gnavus entries refer to distinct strains or to technical or biological replicates of one strain (see Mediterraneibacter gnavus Q-S). The same applies to Bacteroides thetaiotamicron E and F.
The sample selection for the dendrogram representation is less than ideal. It would have preferred if the relationships among cosine distances between (i) replicates of an isolate, (ii) consensus spectra of different strains of a species, (iII) data from different species, (iv ...) etc. were analyzed. In my opinion, the data selection does not allow for systematic conclusions to be drawn: Not all the necessary information is available (see above) and very different taxonomic entities are being compared.

6. I think the focus of the manuscript is too broad. While the abstract suggests that IDBac is a platform for identifying bacteria (see comment #1), significant parts of the manuscript describe other applications, such as new compound discovery using comparative MANs. While I understand the aim is to describe the IDBac platform in its entirety, the enormous breadth of this publication is not helpful. This also causes problems for the reviewers. For example, I do not feel qualified to assess the validity and quality of the NMR data provided.
I also doubt whether the ability of certain species to synthesize secondary metabolites, such as haereomegapolitanin or haereoacidicolin, always correlates with species assignment. In other words, it is unclear whether different metabolite production profiles are necessarily linked to different taxonomic assignments (i.e., species assignments). I think this is unlikely. In my opinion, this topic is a separate story that should not be the focus of the present manuscript.

7. Knowledgebase contribution metadata sheet (Excel Metadata Sheet). In my opinion, the availability of metadata is crucial to the success of IDBac. I am therefore very pleased that the authors have devoted so much attention to this important aspect. However, the task of entering metadata should be automatable. Just imagine how long it would take to enter spectra from 100 isolates!

8. Figure S23. Quality control of protein MS spectra for IDBac-KB depositions. I agree, Quality control (QC) is extremely important. However, I believe it should be performed by applying some objective criteria (please refer to Cuenod et al., 2021; or Lasch et al., 2025). Objective QC saves an enormous amount of time and effort while guaranteeing the comparability of data over a long time scale. After all, no single person (to rule out different – subjective - evaluation standards) will be able to visually assess the quality of data submitted to IDBac. Therefore, QC tools based on “hard” spectrum quality criteria should be implemented. This point could be mentioned and discussed in the manuscript (and perhaps objective QC will become a new workflow in the GNPS analysis environment in the future).

Minor Comments

i. Last sentence of the first text section in “Main” (line 89) Please provide a reference
ii. Second and third text sections in “Main”: My impression is that other solutions for identifying bacteria are not presented in a particularly fair manner. I already pointed out at the beginning that I consider MicrobeNet to be a very easy-to-use, extremely comprehensive, helpful, and well-documented platform. There are also other possibilities to analyze bacterial spectra independently of the databases and software solutions offered by commercial providers: MALDI-UP, MicrobeMS. These solutions should also be mentioned.
iii. Results, line 153. Please describe exactly what consensus spectra are and how they are obtained.
iv. Results, line 183. “Spectra are searched”… As I understand it, spectra are not tested, but peak lists derived from spectra are compared. Please explain.
v. Line 337. Please check “(2)” after haereoacidicolin
vi. Line 376: Personally, I doubt that MALDI-ToF MS is optimally suited to be used for de-replication of strains. For de-replication purposes we made in my lab good experiences with vibrational spectroscopies, i.e., Raman or FTIR spectroscopy, as cost-effective and relatively easy-to-use methods. Compared to MALDI-ToF MS these methods are not that robust, but offer a better taxonomic resolution.

(Remarks on code availability)

Reviewer #3

(Remarks to the Author)
After reading the response to reviewers thoroughly and the manuscript once again, the authors have satisfied the reviewers comments very well. I have no further additions to suggest.

(Remarks on code availability)
I am not an expert in coding and therefore cannot comment on the quality.

Version 2:

Reviewer comments:

Reviewer #2

(Remarks to the Author)
Unfortunately, my attempts to test the IDBac platform using my own data have failed again. As the study's authors will probably be able to see in their server log files, I was again unable to test the IDBac identification pipeline. I followed the instructions in the video tutorials provided very carefully — with the result described.

I would therefore ask the authors to analyze the errors and update the documentation accordingly. While the authors responded in great detail and in a satisfactory manner to most of my comments and suggestions in the second round of review, my final assessment of the submitted study will be based mainly on the success and quality of the analyses of the IDBac workflows. This is primarily because the present study focuses on an online platform for data analysis, IDBac. Although reviewers #1 and #3 carefully evaluated the manuscript, they obviously did not attempt to test the platform itself. It is my view that one of the reviewers should test the IDBac platform before the study is published in the prestigious journal Nature Communications. So this task now falls to me.

(Remarks on code availability)

Version 3:

Reviewer comments:

Reviewer #2

(Remarks to the Author)
NCOMMS-25-72074-T, "IDBac: an open-access web platform to identify bacteria and analyze relationships in culture collections using MALDI-TOF mass spectrometry" by Krull, N.K. et al.
Thank you very much for the detailed feedback. After reviewing the provided explanations and notes, I successfully tested the IDBac pipeline with my own data.
The authors will be able to see from the server log files that I used MALDI-ToF mass spectra from Paraburkholderia caldeonica and a published MALDI-ToF MS dataset containing spectra of highly pathogenic bacteria for the tests. The latter dataset is available at the following link: https://wiki.microbe-ms.com/uploads/ring-trial-RKI-spectra.zip. The corresponding publication is from 2014: https://doi.org/10.1128/JCM.00813-15.
The dataset containing spectra of highly pathogenic bacteria was selected because, while revising their manuscript, the authors had in the meantime integrated mass spectra from the RKI ZENODO Database 4.2 into IDBac. This ensured that the species included in the test dataset are represented in the IDBac database, which I considered a first prerequisite for evaluating the identification methods in IDBac. Second, the fact that the identical RKI spectra are also fully available to both

tools, MicrobeNet and MicrobeMS allowed conducting comparative analyses of identification accuracy between IDBac and the latter two tools.
The selection of MALDI test data for Paraburkholderia caldeonica was based on a similar rationale. The aim was to ensure that spectra for this bacterial species were part of the IDBac database. Paraburkholderia caldeonica was studied earlier in a paper by the Linington group I knew before this review (see https://doi.org/10.1128/AEM.00354-20); some of them are co-authors of the present manuscript.
In general, it can be said that the identification of bacterial isolates at the species level worked well in my tests. Identification was successful with the MALDI test data for Paraburkholderia caldeonica but there were some difficulties when interpreting the data from the test set of highly pathogenic bacteria.
Performing the analyses seemed to me to be time-consuming overall. This is not only due to the need to first convert the MALDI spectra into the mzML format using MSConvert. The metadata must also be entered through a multi-step process that also looked rather complicated to me (though I did not test it).
Overall, I found IDBac to be rather unintuitive. The fact that it took me four attempts just to get the pipeline up and running does not speak well of IDBac's user-friendliness. In my opinion, it would have saved everyone time and effort if the authors had consulted individuals (microbiologists?) outside of the IDBac programming team for testing before submitting their publication. Unfortunately, I had the impression that I was the first external tester of the identification pipeline. During my tests, I often wondered why the analyses stopped at certain points. Clear error messages and progress indicators would have been helpful. With progress indicators or an error message, for example, I would have immediately known that the analysis of a single dataset was not yet complete after four minutes of runtime and that I simply needed to be more patient. The authors shouldn't take this as the grumbling of a disinterested reviewer—I actually do really think that a lot of potential pipeline users will give up after 10 minutes (and never come back) if things don't work right away.
On the other hand, I really liked the range of features in IDBac.
The IDBac standard specifications were used for the identification tests. The results obtained are generally satisfactory. However, the accuracy of identifying the highly pathogenic test set appears to be slightly lower than analyses performed by other free tools, such as MicrobeNet and MicrobeMS. See the appendices for the corresponding analyses and comparison. I can only speculate on the reasons for this discrepancy. One possibility is that using a very wide window of 10 Th for peak detection is not optimal. Furthermore, the test set is rather challenging, due to the presence of closely related species like Yersinia pestis/ Y. pseudotuberculosis, Bacillus species, or species from the Burkholderia pseudomallei group. However, these are precisely the types of situations that tools like IDBac will encounter in routine use.
Unfortunately, I had difficulty determining the species identified based on the file names displayed in the IDBac score lists. Only some of the metadata from the RKI database spectra was carried over during import into IDBac. Therefore, interpreting the score lists required considerable effort. The authors should address the missing metadata issue in a future update.
It would also be desirable if the results of the analyses were presented in a clearer manner. In the default setting, for example, the score lists display all analyzed test dataset results in a single table. Although the display of results can be manually restricted to individual mzML test data, I could not find a setting that allows for a clear presentation of identification results for more than just one test spectrum, as is available in MicrobeNet or MicrobeMS.
Distance estimation using cosine distance appears to work very well. It is an interesting way to further refine identifications based on spectroscopy and/or spectrometry, not only for bacteria.
The dendrograms from the cluster analysis include reference entries from the IDBac database. While this may be helpful in certain scenarios, it would also be helpful to have the option to exclude such database spectra from these analyses and / or visualizations.
On the other hand, I really liked the visualization of the PCA results. The phylogenetic relationships of the species in the test dataset are adequately reflected in the score plots.
QT: Unfortunately, the results of the quality test are not comprehensible. However, since this pipeline was explicitly marked as beta, I will not address these analyses in my review. Nevertheless, improvements of the QT pipeline are definitely required.
Comparable tools, such as the MALDI Biotyper (MBT), MicrobeNet, and MicrobeMS, allow performing identification analyses based on individual MALDI spectra, not only of consensus spectra. When evaluating identification results, it is often useful to be able to analyze replicate spectra of a sample individually. Therefore, the pipeline should offer an option to analyze single MALDI-ToF mass spectra.
As a follow-up to the topic of dereplication: We also gained experience in this area in our laboratory. In various projects, we faced the challenge of how to select phenotypically highly similar isolates from a large number of bacterial samples for subsequent whole-genome sequencing (WGS) analyses. In short, our experience indicates that the taxonomic resolution of MALDI-ToF MS is inferior to that of spectroscopic methods, such as Raman and infrared spectroscopy. With comparable effort in data acquisition and analysis, these techniques enable significantly better differentiation between closely related yet distinct strains than MALDI-ToF MS does. One application scenario for the IR Biotyper, developed by Bruker Daltonics, consequently involves dereplication of bacterial isolates in outbreak investigations. Furthermore, some years ago, RiverDiagnostics, a Raman company in the Netherlands, introduced a corresponding Raman solution for the dereplication in clinical settings.
However, I'm not denying that MALDI ToF MS is suitable for dereplication tasks. However, if I had to choose, I would always use FTIR or Raman spectroscopy for these tasks.
Related literature:
https://doi.org/10.1016/j.ijfoodmicro.2025.111550
https://doi.org/10.3389/fmicb.2026.1735218

Summary: After thorough consideration, it is my estimation that the publication of the manuscript is advisable in its present form. It is my hope that the authors will take my suggestions into account and consider my recommendations when updating IDBac in the future. The initiative to provide free, vendor-independent, and publicly accessible pipelines for MALDI-ToF MS analyses in microbiology, along with the related resources, is very much welcomed. Notwithstanding the presence of certain

outstanding tasks and deficiencies that require attention, it is my conviction that the present manuscript merits publication in its current state.

(Remarks on code availability)

Open Access This Peer Review File is licensed under a Creative Commons Attribution 4.0 International License, which permits use, sharing, adaptation, distribution and reproduction in any medium or format, as long as you give appropriate credit to the original author(s) and the source, provide a link to the Creative Commons license, and indicate if changes were made.
In cases where reviewers are anonymous, credit should be given to 'Anonymous Referee' and the source.
The images or other third party material in this Peer Review File are included in the article's Creative Commons license, unless indicated otherwise in a credit line to the material. If material is not included in the article's Creative Commons license and your intended use is not permitted by statutory regulation or exceeds the permitted use, you will need to obtain permission directly from the copyright holder.
To view a copy of this license, visit https://creativecommons.org/licenses/by/4.0/

Below is a detailed explanation of edits. Reviewer comments are indented, italicized, and in blue font, while our responses are left aligned and in black font. Additionally, we attached a copy of the manuscript with line numbers and major changes in red font, so you are able to compare differences between the original submission and the resubmission. If you require any further material, we would be happy to provide it.

Reviewer #1 (Remarks to the Author):

> *This manuscript presents IDBac, a web-based platform that integrates MALDI-TOF mass spectrometry data—both protein "fingerprints" and specialized metabolite profiles—into an open-access spectral library for bacterial identification and comparative phenotyping. The authors aim to extend MALDI-TOF applications beyond clinical microbiology into environmental and natural product discovery contexts. The concept of bridging microbial identification with metabolomic potential is both ambitious and valuable, and the effort to construct a public, community-driven repository deserves recognition. If implemented rigorously, such a platform could substantially influence how microbiologists dereplicate isolates and assess chemical novelty. While the idea and motivation are commendable, several critical weaknesses prevent the study from realizing its potential. The manuscript currently presents a technically interesting but underdeveloped platform description rather than a mature, validated system with clear biological or methodological contributions.*

First, we would like to thank Reviewer 1 for thoughtful comments. We acknowledge this review likely required significant time investment and we are grateful for this.

Overall response: All major critiques of Reviewer 1 are addressable through further clarification in the text and additional supplemental content. In particular, one major critique – a lack of documentation that our *Paraburkholderia* metabolite was new – was referenced in the main text and provided in detail in the Supporting Information on the initial submission. Data includes full characterization by 1D and 2D NMR experiments and MS/MS analysis. Importantly, the discovery of a new metabolite provides important validation for the platform as a viable discovery tool.

> *1. Library Strain Diversity and Metadata Limitations*
> *The strength of any identification platform lies in the depth, breadth, and curation quality of its reference library. Although the authors have assembled a dataset of approximately 1,400 bacterial isolates, the library appears highly uneven in both taxonomic representation and environmental provenance. Based on Table 1 and the Supplementary Dataset, the majority of entries originate from a narrow range of institutional sources, including the Duchossois Family Institute, NRRL, and a few collaborating academic laboratories. As a result, the dataset is heavily weighted toward a limited set of genera, notably Bacillus and Streptomyces, as well as other Actinobacteria/Firmicutes commonly associated with natural product research.*

Dr. Brian T. Murphy; Phone (312) 413-9057; E-mail: btmurphy@uic.edu

We fully acknowledge that our alliance of government, private institute and academic labs have not procured a comprehensive sampling of existing culturable bacterial taxa. That said, IDBac is currently the largest and most diverse public KB in the world and we have created infrastructure for the community to grow this resource. But lack of a comprehensive KB is precisely ***why*** a crowdsourced approach to repository construction is needed. We stress that the limiting step toward this technology being adapted is the creation of a user-contributed KB and accompanying analysis platform. This point has been emphasized in 9 reviews / perspectives spanning greater than a decade (refs 10, 22-29 in the manuscript).

Since no single research lab or team is in possession of all the world's culturable taxa, the platform must first be created (done), then established through proof-of-concept (done) and publication (in progress), and finally populated by the community (in progress). Two co-corresponding authors on our team, Drs. Wang and Sanchez, were key members of the development team for the Global Natural Products Social molecular networking (GNPS) database that was published in 2016 in *Nature Biotechnology*, which started out with similar numbers of reference spectra and has now grown to 1,850,168 MS/MS spectra in the last decade due to user contributions (2,662 active user accounts, >11,000 unique visitors in October 2025, >4,000 manuscript citations). Following this same model, our team highlights that growing the IDBac KB relies on open access to a consistent and user-friendly graphical interface (GUI).

Re: *Streptomyces* and *Bacillus*: We do concede that these genera are heavily populated in the KB, however outside of clinical pathogens they are some of the most commonly studied and cultured bacteria in the past century. The KB reflects this (~31% and 7%, respectively).

> *This bias is problematic for several reasons. First, it undermines the utility of IDBac as a general-purpose identification tool: users attempting to classify isolates from underrepresented phyla (for example, Proteobacteria, Cyanobacteria, or Bacteroidetes) will likely encounter reduced accuracy or no meaningful match at all. Second, without representation from diverse ecological and geographical origins, the system's capacity to reveal relationships between microbial habitats and metabolic signatures remains constrained. In short, the current dataset provides a promising proof of concept, but not yet a comprehensive or balanced identification compendium.*

Re: Identification accuracy: We agree that limited species entries in the KB will hamper efforts to perform untargeted bacterial ID. This point is addressed by our implementation of a *user-contributed* KB of MS spectra, where the ability of the KB to accurately identify unknown bacteria will improve over time as users upload spectra. As we did with GNPS, we are applying the same proven model to grow the user base of IDBac. As we have shown in the past, publication of the KB is a crucial step toward this goal.

Dr. Brian T. Murphy; Phone (312) 413-9057; E-mail: btmurphy@uic.edu

Re: Growing the user base: We emphasize that many research labs *already possess* culturable isolates that would facilitate accurate bacterial identification (e.g., a lab wishing to target the isolation of genus A typically already has isolates that can be uploaded into the KB and used to accurately ID unknowns from the environment; this has been the field standard for >10 years). A survey of MALDI review articles over the past 15 years shows that most users perform research targeted toward specific taxa and have created local databases of spectra from these taxa (refs 10, 22-29 in the manuscript). But these 'local databases' are currently only useful for the host lab. Our user-sourced model would unite these efforts and 1) encourage past data to be uploaded and 2) ensure that future data are public. This would represent *a major advance* in the field and address the limitation that is consistently brought up in reviews/perspectives.

Our perspective: We wish to argue *against* the need to create a comprehensive KB of bacterial taxa by our team, as opposed to a user-contributed KB. Building a complete KB is not the model that we are proposing and speaking from experience, this is not a reasonable approach toward solving this long-standing problem. Creation of a comprehensive KB of all known culturable taxa is too great of a barrier for one group to overcome. Further, in our opinion (which is the consensus of reviews/perspectives in the field), this is precisely the reason that MALDI technology has yet to be widespread adopted in environmental microbiology, despite its adoption as FDA-approved technology in clinical microbiology for over a decade.

> *Equally concerning is the lack of standardized and complete metadata. Many entries lack detailed contextual information, including source habitat (e.g., soil, marine, host-associated), precise isolation site, and sampling conditions. This omission limits not only the interpretability of spectral clustering but also the scientific reproducibility and downstream utility of the database. Provenance metadata are fundamental for correlating phenotypic variation with ecological or evolutionary patterns. In its current form, the IDBac repository provides little indication that metadata completeness was prioritized or systematically curated.*

IDBac *does* require metadata entry (see **Supplementary File 2**). A standardized Excel sheet accompanies each submission, offering 28 metadata categories and *requiring* 11 of them. However in response to this and a comment from reviewer 3, we further standardized values for the following fields: MALDI instrument, MALDI matrix name, MALDI prep., Cultivation temp., Cultivation time, and Isolate source. While we agree with the reviewer's outlook on the importance of environmental categories of metadata (we even dedicate an entire section to it in the manuscript, lines 229-249), excessive requirements will limit the number of willing contributors to the platform. Therefore we require an NCBI Accession, Genbank Accession, or 16S sequence, in addition to the aforementioned categories (lines 153-157). Completing the remaining categories are strongly encouraged to users of the platform. *Note: For example, not all strains have GPS coordinates, e.g., microbiome derived strains, hence making some metadata categories optional.

Dr. Brian T. Murphy; Phone (312) 413-9057; E-mail: btmurphy@uic.edu

2. Narrative Focus and the Specialized Metabolite Discovery Section

While integrating protein and specialized metabolite profiles under one platform is conceptually attractive, the metabolite discovery vignette currently distracts from the manuscript's core purpose. The primary contribution of IDBac should be its capacity to facilitate high-throughput microbial identification and comparative analysis; however, the inclusion of a "new metabolite discovery" story shifts attention toward an isolated chemical finding that lacks sufficient experimental substantiation.

Comparative metabolic analyses *strengthen* the ability of IDBac to provide phylogenetic resolution during microbial identification (see Fig 5, lines 348-354). As detailed in later responses and in the manuscript text (lines 338-345), the discovery of a new metabolite highlights the platform's ability to use natural product production to compare seemingly identical bacterial isolates; full molecule characterization – which is in the SI and referred to in the main text – is required to validate this approach.

Each section of the manuscript provides a critical proof-of-concept to support the functions within the IDBac Analysis platform. To our knowledge no other platform (public or private) accomplishes this range of analyses and highlighting them all is key to understanding the functions of the platform. The incorporation of the small molecule analyses alongside the database and dendrogram analyses is a major innovation and deviation from existing commercial biotyping options.

<u>Re: lacks experimental substantiation for new metabolite</u>: Evidence to support the fully characterized three-dimensional structure constituted over half of the original Supporting Information provided and consisted of 1D/2D NMR spectra, MS/MS spectra, and Marfey's analysis (**SI Figs. S4, S5, S7-18**, **Supplementary Note 2**, **Table S3**).

According to the manuscript, a distinct m/z feature was detected in a subset of strains and tentatively proposed as a novel metabolite. Yet no structural confirmation, MS/MS analysis, or bioactivity validation accompanies this claim. Without such evidence, the observation remains preliminary—essentially a pattern recognition result. Presenting it as a discovery risks overinterpretation and blurs the distinction between what IDBac can suggest versus what it can demonstrate.

Moreover, devoting an entire section to this anecdote unbalances the manuscript's narrative flow. Readers expecting a methods-centered presentation are suddenly confronted with a tangential storyline that feels incomplete. The result is a paper that tries to tell two stories—database construction and metabolite discovery—without fully delivering on either.

Dr. Brian T. Murphy; Phone (312) 413-9057; E-mail: btmurphy@uic.edu

Re: structure: As discussed prior, our team isolated and fully characterized the three-dimensional structure of the new metabolite haereoacidicolin by NMR, MS, and Marfey's analysis (**SI Figs. S4, S5, S7-18**, **Supplementary Note 2**, **Table S3**).

Re: storyline: Our team employed IDBac to metabolically differentiate two isolates at the species level and this finding was critical toward the identification of a new metabolite that was present in *only one* of the strains. This is a unique and innovative platform feature meant for groups that wish to ID/distinguish strains beyond the level of species, which is critical in the subfield of chemotaxonomy. Further, as pointed out by Reviewer 3, MS-based phylogenetic analysis is a novel application of the platform and a growing trend in the field of bacterial ID – therefore we propose it has high relevance to the manuscript narrative.

> *3. Technical Limitations of MALDI-TOF for Small Molecule Identification*
> *The manuscript exhibits an overly optimistic interpretation of MALDI-TOF's capabilities for small-molecule analysis. While the platform's novelty lies in extending MALDI-TOF beyond the protein fingerprinting range, the technical constraints inherent to the method must be acknowledged with candor.*

We reiterate that this comment is based on the misunderstanding that IDBac was used to characterize the structure of the new metabolite, which is not the case. We added additional text (red) to the Discussion section to emphasize this point (lines 409-411, 413-415):

"Finally, there is often a perception that MALDI MS is a specialized instrument that lags other platforms in mass resolving power and is only meant for proteins due to interference or ion suppression from the matrix peaks themselves…Despite the limitations noted in **Supplementary Note 1**, we have shown that unit-resolution peaks from specialized metabolite spectra greatly aid in dereplication of microbial isolates"

Further, it is possible to achieve accurate mass measurements of metabolites on MALDI-QqTOF, MALDI-FTICR, or MALDI-Orbitrap instruments. This is briefly discussed in the manuscript, however it is beyond the scope of this study. Translation of MALDI-TOF MS/MS methods to query ESI-based metabolite libraries is ongoing work by our team that requires significant methodological development.

> *Resolution and mass accuracy limitations. Standard benchtop MALDI-TOF instruments (e.g., Bruker Microflex, Shimadzu Axima) provide modest mass resolution (500–1000 at m/z 500) and limited mass accuracy. This inherently restricts their ability to distinguish closely spaced molecular ions or isotopic variants. Without high-resolution capability (such as MALDI-TOF/TOF or MALDI-FTICR), any identification based solely on nominal m/z is speculative.*

Dr. Brian T. Murphy; Phone (312) 413-9057; E-mail: btmurphy@uic.edu

Although Reviewer 1 is correct about these limitations, this critique does not accurately represent the work we presented in the manuscript. IDBac is not presented as a molecule identification tool, but we state: "...*how IDBac-Analysis can be used as a hypothesis generation tool in the front end of a researcher's analysis pipeline.*" Wording was added to the manuscript to further clarify this point (lines 413-415):

"Additionally, while MS1 data are currently accepted as input for MANs, IDBac-Analysis is not designed to provide putative compound identifications. The IDBac pipeline is being expanded to include MS/MS specialized metabolite data such as that from QqTOF or Orbitrap-based instrumentation in the near future."

Further, from the original manuscript: "*Here, IDBac-Analysis of MANs was used to prioritize two seemingly identical strains from a collection of 316 for a more in-depth metabolic investigation, where WGS then detailed the biosynthetic differences and the new metabolite was purified and characterized. It is important to note that the MANs provide a direct measure of expression for the metabolites, whereas genome level predictions cannot anticipate the active transcription of a given gene.*" (lines 341-345)

> *Matrix interference. The use of CHCA or similar matrices introduces dense background signals below m/z 700, masking many small metabolites. Even above this range, adduct formation and variable ionization efficiencies produce inconsistent spectra. Since the study employs a single matrix for both protein and metabolite detection, matrix bias could be substantial. Yet this factor is scarcely discussed in the manuscript.*

The IDBac workflow requires a matrix 'standard' on each MALDI plate and matrix peaks are removed from all specialized metabolite spectra. The concern that matrix provides too dense of a background is a common misconception and in practice is not a major problem. We added text to the results section to complement existing language in methodology section (lines 297-298): "Subtraction of a matrix blank is employed to remove *m/z* signals attributable to the matrix."

We acknowledge that select few metabolite signals may be filtered out in this process, but several studies from our team indicate that this does not significantly skew the interpretation of MANs (Shepherd et al, Anal Chem, 2025; Luu et al, Analyst, 2023; Clark et al, ISME Comm 2022; Clark et al, Molecules 2022; Condren et al, Gut Microbes, 2020; Costa et al, J Nat Prod, 2019; Clark et al, Proc Natl Acad Sci, 2018). We added language to **Supplementary Note 2** to further support this point.

Additional studies to support our point: In our experience there is rarely exact overlap with the matrix and analytes of interest. We reported one overlapping small molecule/matrix signal from our studies (4-hydroxytamoxifen; David et al, ACS Med Chem Lett, 2018), where an FTICR mass analyzer was employed to distinguish the overlap from initial measurements on a TOF.

Re: use of a single matrix. Our team recently conducted a comparison of different matrices (CHCA, DHB, 2,4,6-trihydroxyacetophenone (THAP), 1,5-diaminonaphthalene (1,5-DAN), and 5-formylsalicylic acid (FSA)) across a series of bacterial extracts to determine whether the number of features is impacted by the use of different matrices. Though some differences occur, these results have no effect on the accuracy of data presented in this manuscript. Though interesting, this discussion is beyond the scope of the manuscript. As with any user contributed database, it is the responsibility of the user to consider differences in experimental parameters of potential matches/mismatches (eg, differences in matrix).

Lack of fragmentation data. MALDI-TOF profiling, without tandem MS, cannot provide structural confirmation. Thus, any identification or dereplication claim derived purely from intact mass data should be considered putative.

As discussed prior, this point has been addressed (**SI Figs. S4, S5, S7-18**, **Supplementary Note 2**). Our IDBac MANs were not used to suggest structural class, nor do we claim this capability. Text was added to the discussion to emphasize this (lines 413-415). Tandem MS data was collected after regrowing the producing strain in large scale liquid culture. Follow up LC-MS/MS analysis suggested the structural type, and compound purification followed by NMR, MS, and stereochemical analysis determined the structure. This process is well documented in the manuscript and in the SI.

4. Standardization Across Instruments and Reproducibility
A fundamental challenge for any shared spectral database lies in ensuring cross-instrument comparability and reproducibility. The manuscript currently lacks sufficient evidence to demonstrate that IDBac can deliver consistent results across different MALDI-TOF instruments, laboratories, or operators. Without such validation, claims of general utility remain speculative.

Reviewer 1 raises an important point. We conducted extensive statistical analyses and added data and a discussion section to the SI to support that independent of lab of origin, IDBac produced consistent knowledgebase identification results (**Fig. S20-22**, **Supplementary Note 4** and **5**). The data discussed in our manuscript represent results obtained across several users and instruments nationwide, collected on platforms that represent two major MALDI instrument companies (Bruker, Shimadzu), and collected at different periods of time. This information is present in the manuscript (Methods section "*MALDI-TOF MS data collection*" (lines 464-473); **Table S8**).

Dr. Brian T. Murphy; Phone (312) 413-9057; E-mail: btmurphy@uic.edu

Note: Comparison of MALDI spectra across different laboratories is a current norm in the field of clinical microbiology (e.g., Bruker's Biotyper, Biomerieux Vitek MS, etc). However as Reviewer 1 points out it is important to establish cross platform consistency.

> *Data harmonization is particularly critical because MALDI spectra are sensitive to numerous variables: laser energy, matrix crystallization, sample spot uniformity, and calibration accuracy. Even identical instruments can yield subtly different peak patterns, depending on the user's technique or maintenance state. Moreover, the manuscript indicates that data were collected using instruments from multiple manufacturers. While the authors note that spectra were converted to mzML and processed uniformly, there is no quantitative assessment of the effectiveness of these harmonization steps.*

Experiments and discussion supporting the reproducibility and harmonization of data across multiple instruments and laboratories were added to **Supplementary Note 5. Strain Reproducibility**). We thank Reviewer 1, this is an excellent addition to our manuscript.

> *Equally concerning is the absence of a formal reproducibility analysis. The authors do not report whether replicate spectra of the same strain (either technical or biological) consistently cluster together. Without such metrics, it is challenging to assess the platform's reliability for identification purposes.*

We thank Reviewer 1 for the suggestion. An analysis of two *B. Subtilis* 3610 colonies across laboratories and instruments that controlled for cultivation conditions was performed revealing a high-level of spectral reproducibility (standard cosine distance = 0.23, 0.36). Further, additional analysis without controlling for cultivation and MALDI preparation procedures was performed revealing that in a cross-laboratory matching scenario, all nine *B. Subtilis* 3610 strains currently deposited in the KB matched most closely with other *B. Subtilis strains*; 7/9 had a top-1 hit within strain. Further analysis and discussion of this point is included in **Supplementary Note 5** and Supplementary **Fig. S22**.

> *5. Statistical Rigor and Evidentiary Strength*
> *The interpretation of Figure 4 exemplifies a broader issue with statistical and evidentiary rigor throughout the manuscript. The figure—apparently a clustering or comparative visualization—illustrates intriguing patterns, yet the conclusions drawn from it are largely qualitative and lack statistical validation.*
>
> *Suppose the authors intend to show that IDBac spectral clustering reflects taxonomic or functional groupings. In that case, the analysis must be supported by metrics such as PERMANOVA, ANOSIM, or adjusted Rand indices comparing MALDI-based clusters to*

UIC

Dr. Brian T. Murphy; Phone (312) 413-9057; E-mail: btmurphy@uic.edu

16S phylogeny or known taxonomy. Without such quantification, the apparent separation in Figure 4 could reflect arbitrary scaling or sample composition differences rather than true discriminatory power.

Similarly, if Figure 4 highlights differences in metabolite profiles, these observations must be supported by replicate consistency and statistical tests. The manuscript currently lacks any indication that such tests were performed.

Statistical analyses presented in prior responses support our findings and show that IDBac-Analysis is accurate and reproducible across multiple platforms, users, and instruments. Further, we added text to the manuscript (lines 239-245; 258-260), updated **Fig. 4C** (lines 278-284), and added detailed analysis and discussion to the SI (**Supplementary Note 1A, 1B**). This comment strengthened our message. Manuscript text below for convenience:

"Hierarchical clustering revealed homophilic patterns in which samples from within source (lake/sediment) clustered closely (**Methods**). Additional statistical analysis revealed significant differences in dispersion (PERMANOVA: pseudo-F=9.633738, p=0.001, n=79,999 permutations; PERMDISP: F=10.55, p=0.001) and Principal Coordinates Analysis (PCoA) indicated a shift in group centroids between sample sources. Additionally, 16S annotations were integrated highlighting differential genus annotations between sample sources (lake/sediment) in addition to divergence in proteomic profiles within genera (**Supplementary Note 1A**)."

"A significant difference in dispersion was observed (PERMANOVA: pseudo-F = 8.47, p = 0.001, n = 273, 999 permutations; PERMDISP: F = 220.73, p = 0.001) and PCoA was performed, revealing a shift in proteomic centroids between collection time points. (**Supplementary Note 1B**)."

"C. Principal Coordinates Analysis (PCoA) shows multivariate MALDI spectral profiles of each pig carcass isolate, colored by their source timepoint. 'X' symbols represent timepoint centroids. Statistically significant differences in distributions between timepoints is observed (PERMANOVA=0.001, PERMDISP=0.001). The PCoA visually confirms a centroid shift and differences in dispersion with the largest shift between successive timepoints was observed along principal component 1 (PC1) between the 6-hour centroid (PC1: 0.0295) and the 30-hour centroid (PC1: 0.1294). Overall, these findings show the trajectory of spectral profiles over time as bacterial communities change during carcass decomposition."

Reviewer #2 (Remarks to the Author):

I read the manuscript entitled "IDBac: an open-access web platform and compendium for the identification of bacteria by MALDI2 TOF mass spectrometry" with great interest and believe that its discussion of a highly timely and relevant subject will appeal to a wide

Dr. Brian T. Murphy; Phone (312) 413-9057; E-mail: btmurphy@uic.edu

readership. In my view, the manuscript is well written and logically structured. The authors present IDBac, an open access platform which can be used to identify bacteria based on MALDI-ToF mass spectrometry. In addition to this, IDBac represents an analysis infrastructure that offers advanced, non-standard possibilities for MALDI-ToF mass spectrum analysis that enables also the visualisation of specialized metabolite differences.

We thank Reviewer 2 for the assessment, as this was our intention.

Unfortunately, based on the materials provided, I am currently unable to provide a comprehensive assessment of the manuscript. Despite my efforts to register on the IDBac web platform, I have not yet been granted access to the system. I fully understand the authors' decision to restrict the use of IDBac to registered users only, even though comparable online platforms such as MicrobeNet handle this differently. However, either the registration process for IDBac should be simplified, or reviewers should be given trial access to the IDBac platform from the outset.

Without access to IDBac, I am unfortunately unable to provide a qualified assessment of the manuscript. I would therefore be grateful if you could send me the access information for the IDBac web resource. I am really interested in testing IDBac, so once I have received the credentials, I will provide the evaluation of the submitted manuscript promptly.

We apologize for this inconvenience, though upon review of other account requests during this time period we determined the sign-up system was functioning properly. Since only submitting new tasks requires login credentials, for the reviewers' convenience, below we included publicly accessible links for IDBac Analysis associated with main text Figure 3:

Workflow: https://gnps2.org/status?task=8e0cb0c6a3c04ae1991bbc1dca2882b5
Interactive Application: https://analysis.idbac.org/?task=8e0cb0c6a3c04ae1991bbc1dca2882b5

Additionally, we have provided login credentials below, so that they may assess the platform in more depth if they wish. Login at the following link (GNPS2 hosts IDBac workflows): https://gnps2.org/homepage

Reviewer 1:
- Username: idbac_reviewer1
- Password: VOE&VfKM&Wq4nQ4D

Reviewer 2:
- Username: idbac_reviewer2
- Password: rAk6Ew3F91C!&!#y

Reviewer 3:
- Username: idbac_reviewer3

UIC

Dr. Brian T. Murphy; Phone (312) 413-9057; E-mail: btmurphy@uic.edu

- Password: GmVD714d$eGpU0e3

Reviewer #2 (Remarks on code availability): The provided link to Github did not work - page not found - 404 error? Authors, please check the link.

We thank the reviewers for taking the time to look into the codebase and we apologize for the inconvenience. We were under the understanding that release of code was not required prior to publication and have now opened the repositories. Prior to publication, code will be deposited in Zenodo.

Reviewer #3 (Remarks to the Author):

The manuscript describes the web platform rollout of IDBac, a tool that has existed for some time now, and this interface significantly increases the usability and user experience. I think the authors have provided sufficient novelty from previous manuscript detailing IDBac and the online interface makes this tool more accessible and FAIR. Like much of the work in natural products research, borrowing on the medical community advances, IDBac seems to provide users with the ability to rapidly ID strains, nullifying some of the research expense that comes with sequencing. One could argue that the necessity for the instrumentation is equally a limitation for those researchers outside of North America, Europe, and Asia.

We thank Reviewer 3 for the assessment, as this was our intention.

Two questions regarding the tool and workflow, (1) is metadata curation done under controlled vocabulary, to enable more efficient mining of the repository. This is an essential step in ensuring interoperability across the database, making future use by you as the creators and the users much more efficient for providing statistics and re-analysis.

We strongly agree with the reviewer's position on metadata curation and note that it is difficult to maximize information richness while preventing barriers to data contribution. As discussed in response to Rev. 1, we required metadata input for essential categories (e.g., MALDI matrix, cultivation media, cultivation temperature, etc) and recently added standardized columns to reduce variance in user responses: *MALDI matrix name*, *MALDI prep*, *Cultivation temp*, *Isolate source*, *cultivation media*, *Instrument model* (**Supplementary File 2**). However, given the breadth of possibilities for additional experimental parameters, further standardization via a controlled vocabulary is challenging. As this resource continues to grow, the list will be updated accordingly.

Dr. Brian T. Murphy; Phone (312) 413-9057; E-mail: btmurphy@uic.edu

(2) While many MS-based tools have little QC, something like this that could be used to ID strains maybe should have some level of QC, especially if users may use it in context with pathogenic strains where IDs may shift perception of required biosafety levels. Is there a precedence from clinical biotyping to standardizing and determine data quality, especially across labs? Or is this something that could be developed in the future (addition of specific inexpensive standards that are then QC checked by the IDBac system, without which data is rejected?) I don't think these hold back the current advancement but enhance the analysis and should be considered for the future.

We agree with Reviewer 3 on the importance of QC and uploading quality spectra into the IDBac Analysis platform. This was addressed in **Figure S20** and in the manuscript (lines 382-385): "*IDBac offers a 'dry run' deposition with integrated spectra visualization that allows users to manually inspect spectra before contributing to the public-facing database. Additionally, we have provided detailed guidance on best practices for MALDI-TOF MS sample preparation and QC (see **Data Availability**).*"

Additional infrastructure is provided on IDBac admin dashboards (https://idbac.org/admin/qc) that enable the rapid screening of large portions of the database for poor spectral quality. Further, the inclusion of user IDs, user names, and principal investigator names ensures that spectra remain attributable to labs and users.

Regarding standardization, we appreciate the reviewer's suggestions. While we are aware of the inclusion of biological standards for instruments (e.g., AXC115), spectra for these standards are not currently included in the IDBac Knowledgebase. We hope to include such standards should they become readily available. As a more cost-effective option, we encourage the use of protein standards for instrument calibration; this is standard operational procedure for any MALDI instrument owner. We added this reminder to the IDBac user guide.
(https://sites.google.com/uic.edu/idbac-documentation/).

I believe the analyses have covered a wide breadth of applications and show the power of utilizing such a workflow, especially showing the timescale study in Figure 4C. Do you have % variability explained for this plot?

Based on a similar comment from Rev. 1, we replaced the PCA (18.21% variability) in figure 4C with PCoA (19.08% variability) (**Supplementary Note 1B**, lines 278-284).

For the MANs, I think this is a valuable asset to now have integrated into the IDBac system. It builds from the vast knowledge of chemotaxonomy that has existed for some decades in plants and fungi and I think you need to add some of this context to the manuscript. There are multiple reviews that exist and I will let the authors decide what is most appropriate. I also believe the MANs are slightly reflective of a preprint describing

metadata-based molecular networks (https://chemrxiv.org/engage/chemrxiv/article-details/6687b32d5101a2ffa875e356) and I think you have to acknowledge the existence of this development, as it mirrors mapping features on selected metadata groups (in your case phylogenetic metadata).

We thank Reviewer 3 for these comments, we added chemotaxonomic context to the manuscript and referenced the preprint in addition to a recent review (lines 231-232). The updated text is as follows: "Further, the integration of metadata to enhance large metabolomics analyses has been performed, though cross-user comparisons remain a challenge in the field for a variety of reasons."

The section from lines 298-309 is effective but more a reflection of the poor quality of 16S to distinguish species (which is widely acknowledged for genera with many species) than the distinguishing power of metabolomes to differentiate species, again leaning on chemotaxonomy as an entire field. I believe this could be reframed in an explicit way, 16S fails us and WGS is expensive on larger scales, therefore using metabolomes to differentiate species is an efficient means similarly to proteomics. Does your proteomic data reveal a difference between these two species as well or do you only get this taxonomic differentiation with the metabolomics? I believe in regards to this assessment, the subheading title needs to be changed to species discrimination, as the two Paraburkholderia were never the same species in the first place.

We agree with Reviewer 3. Just after this passage we added additional language to emphasize the point: "*This discovery highlights the shortcomings of 16S rRNA sequencing to distinguish bacterial strains. It also stresses that although WGS remains an effective way to identify and detail differences between phylogenetically similar bacteria, it is not always a cost-effective means to screen for differences in a large collection of isolates. Here, IDBac-Analysis of MANs was used to prioritize two seemingly identical strains from a collection of 316 for a more in-depth metabolic investigation,…*" (lines 338-342).

Additionally, we've altered the subheading to "*Bacterial species discrimination*…" (line 306). To their question as to whether proteomic data also distinguishes the two strains: the protein MS profiles are highly similar, though not identical, thus further highlighting the power of the MAN to provide an orthogonal level of chemotaxonomic differentiation.

In figure 5A, highlight where in the MAN compounds 1 and 2 are, this reinforces the technology.

This comment was addressed in response to Rev. 1. Text was added to clarify that we do not claim to observe/identify any molecules from the MAN alone, as this would be beyond the resolving power of the instrument (lines 413-415). While *m/z* = 764 ($[M+H]^+$ haereoacidicolin) does

appear associated with all 1-D8 replicates, it was filtered from the MAN visualization due to low intensity. Most importantly, the MAN in Fig. 5A highlights that the two proteomically similar strains are metabolically different. As we describe in that section, this observation was used to prioritize these strains from a large collection of bacteria for more in-depth analysis.

Structure elucidation is well done, you should deposit the NMR data into NP-MRD (https://np-mrd.org/) if not done so already. NMR data in supplementary essential as screenshots are not very useful and this repository makes it more accessible.

We thank the reviewer for this suggestion. Data were uploaded to NP-MRD (https://np-mrd.org/natural_products/NP0351721). This was added to the manuscript in the Methods section (lines 633-634).

Reviewer #3 (Remarks on code availability): I am not an expert in coding and therefore cannot elaborate on its quality.

Dr. Brian T. Murphy; Phone (312) 413-9057; E-mail: btmurphy@uic.edu

We thank the editor and reviewers for their thoughtful review of our revised manuscript. We were excited to see that major concerns from Reviewers 1 and 3 were addressed in our revised submission. Below is a detailed explanation of edits to address Reviewer 2's critiques. Reviewer comments are indented, italicized, and in blue font, while our responses are left aligned and in black font. Additionally, we attached a copy of the manuscript with line numbers and major changes in red font, so you are able to compare differences between the original submission and the resubmission. If you require any further material, we would be happy to provide it.

Reviewer #1 (Remarks to the Author):

The manuscript has been revised extensively and has improved considerably. The authors' point-by-point responses are appropriate and resolve the issues raised. I have no further comments on the revised manuscript.

Reviewer #3 (Remarks to the Author):

After reading the response to reviewers thoroughly and the manuscript once again, the authors have satisfied the reviewers comments very well. I have no further additions to suggest.

Reviewer #2 (Remarks to the Author):

NCOMMS-25-72074-T, " IDBac: an open-access web platform and compendium for the identification of bacteria by MALDI TOF mass spectrometry" by Krull et al.

In this work, Krull and his colleagues introduce a web-based platform, IDBac, which is described to allow not only identification of bacteria based on their MALDI-ToF mass pattern, but also probing taxonomic relationships between them, and visualizing MALDI differences of secondary metabolite signatures produced by closely related bacteria. To this end, the authors have created a reasonably comprehensive MALDI-ToF spectrum database and present in their manuscript various tools for spectrum analysis and visualization. This ambitious undertaking is intended to be expanded in the future with data from external users and represents a potentially interesting extension of analysis options for more than just MALDI-ToF-MS identification of bacteria.

First, we thank Reviewer 2 for re-reviewing our manuscript and experimenting with the platform itself. They invested a significant amount of time doing so and they are well-versed in the current standards of bacterial identification by MS. There appears to have been a small misunderstanding with the types of data files that are accepted by our platform which we will address but want to thank them for providing an excellent and thorough review. We made

several revisions that both clarified the story flow and improved the usability of the platform. These are detailed in our responses below.

I very much welcome the idea and motivation behind the project, but I must note that the manuscript does not appear to be sufficiently focused in its current form. It is my general impression that the authors are more interested in including and presenting a high number of functions and methods for analyzing bacterial MALDI spectrum data rather than on usability and simplicity.

This not only detracts from the clarity and comprehensibility of the manuscript. Additionally, the IDBac (or better the GNPS2) web platform appeared to me somewhat confusing. In my opinion, users without an extensive IT background will only be able to use it after a lengthy training period. Both the manuscript and the IDBac platform could benefit from focusing on a few, selected core functions.

My comments and criticisms are detailed below.

Re: platform usability. We thank the reviewer for revealing potentially confusing portions of the IDBac platform, especially in the context of the significantly larger computational GNPS2 platform on which IDBac was built. We have made significant improvements to the usability of the platform to address this concern. Though as we will detail in our response to critiques below, our records indicate that Reviewer 2 appears to have uploaded the incorrect file type into the IDBac Analysis portal, which would prevent meaningful analysis (ESI files as opposed to MALDI MS files; this was not part of our platform protocols). This likely contributed to a perceived negative user experience with the platform. To better prevent this for new users, we constructed a prominent "Introduction & Video Tutorials" link on the main page that links to detailed video instructions for each step of the data conversion and analysis process, found here. These video tutorials will help facilitate user adoption and are detailed in this response document. Additionally, further data validation steps were added to the workflow to provide more informative error messages for future users. Finally, we have revised the idbac.org portal to provide direct links to the appropriate workflows in GNPS2 without having to wade through the other 70 non-IDBac relevant workflows in the platform.

Re: manuscript focus. In its current form, the manuscript focuses on the three main functions of the IDBac platform: 1) bacterial identification via the IDBac-knowledgebase (IDBac-KB), 2) metadata contextualized comparison of bacterial isolate similarity, and 3) use of metabolite association networks to distinguish closely related bacterial strains. Although bacterial identification is a major function of the platform, the two additional ID-related functions are equally essential to highlight because IDBac *is* more than just a database and that it is different from existing biotyping platforms. IDBac has potential to not only ID bacteria, but also compare bacteria through a variety of innovative analyses. The additional two

functions are particularly critical for labs that wish to compare large numbers of bacterial isolates for analysis and/or prioritization, which is a common bottleneck across the microbiological research sphere and to which few options to do so currently exist.

As self-identified, Reviewer 2's research interests lie in Function #1, use of IDBac to identify bacteria, therefore their desired manuscript focus is understandable. We accommodated Reviewer 2 by better framing the IDBac story. We added a more balanced presentation of the platform that replaced our heavy emphasis in the beginning of the manuscript on bacterial ID using the public database. First, we made a minor revision to the title of the manuscript to better manage reader expectations (changes in bold):

> "IDBac: an open-access web platform to identify bacteria and analyze relationships in culture collections using MALDI-TOF mass spectrometry."

Second, we modified the abstract to more accurately represent manuscript and knowledgebase content. We agree with Reviewer 2 that as framed in its prior form, additional platform functions presented in the manuscript seemed to broadly expand the story, particularly since the previous abstract focused predominantly on bacterial ID. This gave the impression that the major focus of the manuscript was the KB. However, as we show in the manuscript, IDBac was designed with the unique ability to analyze large collections of bacteria in bulk.

Third, we organized the presentation of the IDBac platform into the three ID-related "Features" listed in the prior paragraph (bacterial ID, metadata analysis, metabolite association network analysis). "Feature 1", "Feature 2", "Feature 3" were incorporated into section headings in the manuscript that now help better organize the material for the reader and manage their expectations.

> *After the authors provided me with the login details for the IDBac platform in response to my request in the first review, I was able to log into the platform as idbac_reviewer2 using the credentials supplied. This worked quite well, but unfortunately my testing were unsuccessful. My original intention was to challenge the IDBac spectrum library by own spectrum data (BrukerRaw format) from selected Paraburkholderia species from which I knew they are represented in the IDBac database - after all, the authors had raised my expectations to do so by the very first sentence of their abstract: "The identification of bacteria is central to the microbiological sciences"*
>
> *I do not want to go into all the details of my test here, but I found that the IDBac-Analysis workflow is just one out of 70 workflows hosted on the GNPS2 Analysis Hub website. Furthermore, I learned that potential IDBac users must first convert their spectrum data according to what seemed to me to be a rather complex scheme. I*

might have achieved a positive result if I had invested more time in testing IDBac and/or used the IDBac support.

A major barrier to the development of community resources is the creation of a platform with long-term support, capable of supporting multiple synchronous users that have computationally intensive analyses and large-scale data. The computational workflows that underlie IDBac are hosted on GNPS2 infrastructure, a partnership that has enabled us to scale IDBac as a free resource for the academic community. It provides zero-cost hosting for web services and access to a high-performance computing cluster backed by 350+ CPU cores and 600+ TB of user storage. To help guide users to IDBac-relevant workflows we have included direct links to them (with links to accompanying documentation) at idbac.org.

Additionally, as Reviewer 2 acknowledges, the conversion to an open-standard file format designed by the Proteomics Standards Initiative under the Human Proteome Organization adds an additional step to our pipeline. We concede that a platform that accepts only one file type is easier to use, however this option is most suitable for commercial platforms and reflects one of the current challenges in the field of MS analysis, namely multiple instruments from multiple vendors. The file conversion step facilitates the use of IDBac by users of *any* instrument platform and ensures our adherence to NIH Data Availability guidelines and our groups' policies of maintaining FAIR (Findable, Accessible, Interopable, Reusable) standards in our published work. File conversion is always a barrier and we chose to integrate it into the platform and strongly recommend users to follow the detailed instructions provided. To accommodate this barrier, we created a readily accessible ~3 minute video tutorial that walks users through file conversion (link here).

However, compared to MicrobeNet, a similar solution for the taxonomic analysis of MALDI-ToF mass spectra that I use regularly, I found that IDBac has some weaknesses in terms of efforts, user-friendliness, and usability. Although the manuscript points out several times that IDBac has a significantly larger range of functions than alternative solutions, probably MicrobeNet is meant, it left me with the impression that using IDBac requires in-depth prior knowledge. The GNPS2 Analysis Hub indeed offers many different, potentially useful functions. However, as a user seeking to identify bacteria based on their MALDI pattern, I found the website rather confusing and did not always understand the purpose of the workflows hosted.

Re: advantages of MicrobeNet. MicrobeNet provides rapid and effective identification of predominantly *pathogenic* microbes based on MALDI-TOF data collected only on *Bruker instruments*. For users who fit this, we agree, it is an effective bacterial ID option and the narrower scope of MicrobeNet allows for a streamlined experience. However, for our target audience (researchers not working with pathogenic bacteria) these are two significant user limitations, as documented in the introduction of the manuscript (L93-101). IDBac provides a

wider range of analyses, utility to other instrument vendors, and reference spectra beyond the clinical domain. These were all major barriers to creation of diverse, community-contributed platform. The conversion of files to open file format is required and inevitably the resulting functionality can appear complex for the first time. We created video tutorials to help mitigate this. But we stress that as of now, this is necessary to achieve the platform's flexibility. Historically within the metabolomics community a decade ago, we ran into similar problems with the development of the original GNPS platform. We followed a similar path of education and tools to make conversion significantly easier. Today, within the LC/MS metabolomics community, conversion to open formats no longer has become an issue with GNPS/GNPS2 having analyzed over 30M unique LC/MS samples from tens of thousands of users in the community.

RE: user-friendliness. In order to increase the visibility of and interaction with the IDBac user protocols, we embedded a series of short video tutorials to the IDBac documentation including overviews of each of the three major IDBac functions ("Identify bacteria", "Integrate metadata", and "Assess metabolite overlap"; link to all three). We embedded additional videos on the documentation page that walk users through the data preparation and analysis process step-by-step: "Prepare files for IDBac", "Contribute to the Knowledgebase", and "Analyze data". Additionally, with successive feedback from users, we will continue to simplify the file conversion and upload process.

We do sympathize with Reviewer 2 that MicrobeNet requires fewer front-end steps, however as mentioned that module is designed to accept only files produced by a single vendor, which is an inherent limitation and barrier to use.

> *Unfortunately, my attempts to perform analyses using sample files stored on the website (spectrum data from the authors or their cooperation partners) were unsuccessful as well. Since no error messages were displayed, I stopped trying to use the IDBac website after about 90 minutes. However, I will not blame the authors for this failure in this review. It is possible that I overlooked or did not understand all relevant aspects. Nevertheless, the authors should bear in mind that their offering is essentially aimed at microbiologists who want quick results with minimal time and effort. In my opinion, IDBac's success will depend mainly on the user experience when visiting the website for the first time, not on the range of functions offered.*

We investigated the errors reported by the platform in the anonymous reviewer workflows and determined that Reviewer 2 selected electrospray ionization (ESI) data from public repositories, rather than MALDI data. In order to prevent other users from attempting this in the future, we added an automatic verification check to the platform to verify the data is from a MALDI instrument. If the reviewer would like to run a workflow using sample data, they can use this URL with pre-filled fields. They must first sign in to GNPS2 in order to access the workflow.

We agree that IDBac's *initial* success will depend on some users' first time experience. We hope the new series of short (1-3 minute) step-by-step videos will reduce the initial barrier to use. However in our experience developing community bioinformatics pipelines, widespread use is achieved slowly after the following steps: 1) publication of the platform in a reputable journal; 2) offering continuous hands-on pre-conference workshops (e.g. Gordon Conference, ASM, ASMS, SIMB, etc); 3) demonstrating the platform's abilities through several application-based publications from other users. Despite learning pains with any new technology, these factors will slowly convince potential users of the benefits of investing time to learn a slightly more labor intensive platform.

Major Comments

1. Abstract, lines 60-61. The phrase "… would facilitate strain identification" should be replaced by " … would facilitate identification at the species and in some instances at the subspecies level".

I do not support the opinion that MALDI-ToF MS is a tool that generally allows strain identification. In my experience, contrary to a number of over-optimistic studies, MALDI-ToF MS only shows limited taxonomic resolution. MALDI-ToF MS has a taxonomic resolution that is limited to the species level, unlike methods such as WGS, FTIR, or Raman spectroscopy. Sometimes, it cannot even differentiate between related microbial species. For example, MALDI-ToF MS cannot reliably distinguish between selected B. cereus group species, E. coli and Shigella, or Citrobacter freundii complex species. To avoid misunderstandings, it should be noted that the latter statement does not mean that MALDI-ToF MS can never distinguish between different strains, biovars, or subspecies within a species.

We apologize for the confusion, the spirit of this sentence was "isolate identification" and we have since swapped "strain" for "isolate" to disambiguate these two meanings. We generally agree with Reviewer 2's sentiment and acknowledge that MALDI-ToF MS has known limitations regarding specific strain identification. We note these in the manuscript (l389-402).

We hope that this addresses the reviewer's concerns about providing *strain* identification, though we wish to highlight that some in the field have begun to push the traditional boundaries of MALDI past the level of species distinction and employ the instrument to effectively distinguish between some bacterial genera. This function may be more applicable to the environmental microbiological sciences as opposed to the clinical sciences. However, as we wrote in a recent publication, this research topic is in its relative infancy and requires many more studies to understand the biomarkers that drive MALDI dendrogram groupings. The IDBac-Analysis platform provides a central location for microbiologists of all sub-disciplines to study this in depth and work towards understanding and minimizing these limitations over the course of several years.

2. The results on line 159 state that "IDBac represents the largest and most comprehensive open-access MALDI-TOF protein knowledge base to date." This statement does not correspond with the facts. The MicrobeNet database, for example, is also openly accessible and can be used without registration, though they require Bruker-formatted XML data files for testing. For instance, MicrobeNet's CDC databases (CDC, CDC Tick, and CDC Unidentified Bacteria) contain a total of 2,240 MSPs: 1,647, 501, and 92, respectively. Second, the RKI database of highly pathogenic bacteria, which is freely available on ZENODO (Lasch et al., 2025), has 1,599 strain entries, exceeding IDBac's 1,418. The EMALiMB database (Ban et al., 2025; RIKEN Institute, Japan) contains 1,736 MSPs. The MALDI-UP spectrum catalog, a user spectrum exchange platform based in Stuttgart, Germany, lists 8,565 entries (version 2026-01-13) from a variety of different taxa. Finally, MALDI-ToF data collections such as MARISMA (Schmidt-Santiago et al., 2024) and DRIAMS (Weiss et al., 2022) contain gigabytes of well-characterized bacterial mass spectra of clinically relevant pathogens for AMR profiling (DRIAMS contains 145 GB of raw spectrum data!).

Thank you for drawing our attention to this point. We would like to draw a distinction between publicly available "analysis platforms" and publicly available "spectral databases". Specifically, while Microbenet is a publicly accessible analysis platform, raw spectral data associated with CDC and Bruker databases in the Microbenet environment *are not available* for public download and the spectral database itself should not be considered open-access.

Similarly, while we agree that MALDI-UP is a promising resource, we note that most spectra *are retained by individual users* and in most cases only spectral metadata and user contact information is available to the public (https://maldi-up.ua-bw.de/questions.asp). We intend to reach out to the organizers of MALDI-UP and see this as a promising source of collaboration for future work.

Regarding larger publicly available spectral databases, this misunderstanding is a matter of defining "open-access". We adjusted our language to clarify that we mean "open-standard format", and we have added content to highlight the benefits of our spectral database relative to publicly available datasets. We compiled this into a table (see below and in manuscript Table S1).

Finally, since re-submission we integrated the RKI database into our platform and noted this in the manuscript text (l163-166; also see text below). This raises the total knowledgebase composition to 3,399 entries and 166 genera. We are aware of other large-scale databases and are working to integrate them into the KB. The DRIAMS database, though substantial, lacks adequate disclosure of treatment of replicates and therefore we have decided against including it in our database. IDBac requires multiple technical replicates per sample to ensure reproducible peak profiles.

	Resource	Bacterial identification (Feature #1)	Integrated metadata analysis (Feature #2)	Specialized metabolite analysis (Feature #3)	Web-based analysis	Provides open-standard file formats	Accepts interoperable file formats
Tools + Repositories	IDBac-KB	✓	✓	✓	✓	✓	✓
	MicrobeNet	✓	✗	✗	✗	✗	✗
	MicrobeMS	✓	✓	✗	✗	✗	✓
Strictly Repositories[1]	MALDI-UP	✗	✗	✗	✗	✗	✗
	RKI[2]	✗	✗	✗	✗	✗	✗
	EMALiMB[3]	✗	✗	✗	✗	✗	✗
	MARISMA[4]	✗	✗	✗	✗	✗	✗
	DRIAMS[5]	✗	✗	✗	✗	✗	✗

1. Table includes only some of the major repositories intended to be a resource for researchers, as opposed to the many available more narrowly focused collections of data that contain few specific taxa (e.g., Vibriobase).
2. Robert Koch Institute (RKI)
3. Expanded MALDI-TOF MS Library for MALDI Biotyper ® (EMALiMB)
4. MALDI-TOF Acquisition Repository of Infectious Species at Marañón (MARISMA)
5. Database of Resistance Information on Antimicrobials and MALDI-TOF Mass Spectra (DRIAMS)

* Other available analysis tools (eg, SPeDE, which compares spectral similarity of different MALDI samples) were not included in this table as they do not have an ID function, do not contain a repository, or both.

Table S1. A comparison of several currently available analysis platforms and spectral repositories. Existing large-scale MALDI-TOF data repositories typically consist of vendor specific formats primarily including .btmsp and 'fid'. In contrast, IDBac-KB uses the open-standard .mzML file format that enables broad interoperability between instrument vendors.

"IDBac represents a substantial contribution to open-access MALDI-TOF MS protein spectral repositories. At the time of publication it consists of 1,418 entries that comprise 6 bacterial phyla, 72 families, 151 genera and 472 species (**Fig. 2**) and to our knowledge is the largest open-standard format spectral database to date . These numbers exclude public datasets that were incorporated into the KB. The integration of version 4.2 of the Robert Koch Institute Database of Highly Pathogenic Microorganisms substantially augmented our efforts and increased coverage to 3,399 entries, 8 phyla, and 166 genera. We note, however, that analysis/results presented in this manuscript do not include strains from the Koch database."

Apart from these figures, it should also be noted that the IDBac database is dominated by strains and spectra mainly from Actinomycota. For instance, Streptomyces, Microspora, and Bacillus account for over half of all IDBac entries (see Figure 2B). (Figure 2B).

Therefore, please remove the misleading statement in line 159 (and all other instances) from the manuscript.

We thank the reviewer and adjusted this and similar statements to more accurately reflect the composition of our database (see red text in previous response). And whereas we do concede that Actinomycetota (in addition to *Bacillus* spp.) are heavily populated in the KB, outside of clinical pathogens they are some of the most commonly studied and cultured bacteria in the past century. The KB reflects this reality.

3. Cosine distance (on a 0-1 scale, 0 signifies identical). To me it is not clear how exactly the calculations are performed and why the cosine distance and not some other distance measure is preferred. What exactly are the advantages of this type of distance over other distances like Euclidean, Hamming, Manhattan, correlation-based distances, etc.?

This is an excellent point. We added the paragraph below to the manuscript (l415-420). The focus of this work on "Cosine" and "Presence" metrics is driven by our successful experiences in the original desktop version of this platform (PMID: 29686101) and recent literature (PMID: 31506264). We note that the workflow does support additional distance metrics (e.g., Euclidean distance) and we are happy to introduce new metrics at user request.

> "Fourth, currently the mechanism of grouping isolates within IDBac is limited to three distance functions (Cosine, Presence, Euclidean), which is a subset of existing literature. There is no distance function that is universally most effective at grouping bacterial isolates; each function has advantages and limitations. We caution, however, that spectral distance is both a function of the distance function itself and how the raw spectra are processed into peak lists. Therefore, to the best of one's ability each experimental dataset should be scrutinized accordingly. Additional distance metrics will be tested as the database continues to expand."

I also consider it a disadvantage when a universal threshold cannot be defined (cf. line 192: "there is no universal threshold to define genus/species boundaries between taxa"), as is the case with the significantly more complex score values in Bruker's MALDI Biotyper (MBT). The MBT defines such general thresholds, and the identification results are color-coded according to a traffic light scheme. This has major

advantages for end users, but admittedly does not always work consistently in the MBT either.

We agree that this is a limitation of the current work, though as the reviewer notes, the entire field of MALDI bacterial ID shares this limitation. In our opinion this is one of the next big frontiers in the field, the exploration of how genus-level dendrogram cut heights vary by taxa.

When analyzing the dendrogram of Figure 3 it appears to me that cosine distances are perhaps not optimally suited for discrimination of different bacterial taxa. The dendrogram given suggests that some species are discriminated at distances ~0.80 (e.g. some Bacterioides) while phylogenetically much more different taxa like Mediterraneibacter (Class: Clostridia) and Lactococcus (Class: Bacilli) are separated by a similar cosine distance of 0.81. Therefore, I do not consider this characteristic of the dendrogram to be particularly advantageous.

We thank the reviewer for raising this point, however we would like to draw a distinction between KB-query cosine distances and proteomic profile clustering analysis using dendrogram cut-heights, as they are two different approaches for data visualization. Each of the microbiome isolates in question was queried against the IDBac-KB; cosine distance scores to their nearest matches are listed in Supplementary Table S2. This analysis resulted in the successful identification of 10 out of 18 query isolates using a threshold of 0.65 distance. Importantly, a cosine distance in this context is limited to similarity between a pair of isolates and does not take relationships across the entirety of a dataset into account.

To accommodate research objectives that require individual bacterial identification *and* collection-scale analyses, protein dendrograms are offered, where hierarchical clustering is applied in conjunction with cosine distance (as depicted in **Fig 3B)**. This allows for visualization of KB matches while providing insight as to how multiple query strains relate to each other at a dendrogram cut-height.

With regard to the mentioned Bacteroides example, the dendrogram does join at 0.8 distance forming a uniquely Bacteroides cluster. However, this distance far exceeds the assigned 0.65 threshold and is too high for confident species or genus-level assignments (though within that grouping one can have higher confidence in the assignments at 0.68, 0.61, etc). As discussed in lines 221-227, when applying hierarchical clustering to consensus spectra of ionized proteins, it is common to see members of a given genus distributed throughout a dendrogram. The dendrogram is not a phylogenetic tree. In this case of Bacteroides, we see members of the same genus 'next to each other', *but the cut-height does not imply a high degree of similarity*. Instead, it shows divergence of protein MS signatures between members of the *Bacteroides* isolates in the dataset. This phenomenon likely drives many sub-species level distinctions in MALDI analysis. This divergence is also observed in the *Lactococcus* example noted by Reviewer 2.

However, these variable thresholds do not necessarily have to be based on the distance metric used; rather, they could be attributed to the type of preprocessing applied or the cluster analysis method used. So, please explain exactly how the data was preprocessed for hierarchical clustering. Similar to what is described starting at line 505? In this context, it is important to know if peak tables were compared as assumed (as it is done with the MBT/Bruker). If so, how many peaks were selected from each spectrum? Was it ensured that the number of peaks per spectrum corresponded to a specified number?

We have revised the methods section to more precisely describe how clustering analysis was performed:

"The interactive protein dendrogram, implemented with Plotly and SciKit Learn Python packages, allows users to explore different approaches toward hierarchical clustering of protein isolates (e.g., average, single, and complete linkage). Specifically, the workflow applies the user-specified pairwise distance function to generate clustering analysis based on processed consensus spectra (see **Methods: IDBac-Analysis Workflow**). Additionally, it allows users to specify the number of database hits by both distance and number, allowing for a focused analysis of top candidates."

To provide additional context, the same peak-picking and distance function that is used for database search is employed for an all-pairwise comparison of query strains and retrieved database strains. These distances are then used for the downstream clustering analysis.

Re: peak picking. For any given spectrum, the number of peaks will vary. Peaks that meet the detection threshold will be kept. Current distance functions (cosine, presence, and manhattan) implicitly penalize significant differences in peak count between two spectra.

4. The complete list of strains should be made available at the IDBac / GNPS2 Analysis Hub website.

The IDBac website provides this information. All strains and their associated metadata can be found at the following URL: https://idbac.org/knowledgebase.

5. Dendrogram of Figure 3. Unfortunately, the strain names are missing, so it was unclear to me if the different Mediterraneibacter gnavus entries refer to distinct strains or to technical or biological replicates of one strain (see Mediterraneibacter gnavus Q-S). The same applies to Bacteroides thetaiotamicron E and F.

The sample selection for the dendrogram representation is less than ideal. It would have preferred if the relationships among cosine distances between (i) replicates of

an isolate, (ii) consensus spectra of different strains of a species, (iII) data from different species, (iv ...) etc. were analyzed. In my opinion, the data selection does not allow for systematic conclusions to be drawn: Not all the necessary information is available (see above) and very different taxonomic entities are being compared.

Re: missing strain names. All unblinded query IDs of individual microbiome isolates are listed in Supplementary Table S2.

Re: clarification of replicates/consensus spectra critique. We added text in lines 206-208 to clarify that microbiome-associated spectra were processed as described in **Methods** and consensus spectra of each isolate were queried against the IDBac-KB. Importantly, the use of consensus spectra removes the need for individual replicate analysis as peaks must be present in >50% of replicates to be retained. This minimizes variance that may occur during data collection.

> "IDBac-Analysis supports high-throughput taxonomic annotation with batch MALDI MS acquisitions. As a demonstration, we performed a blind analysis of 18 in-house isolates from the human gut microbiome. Consensus spectra (representing all replicates of each isolate) were simultaneously queried against the IDBac-KB using a cosine distance threshold of 0.65 (see **Methods:** MALDI-TOF MS target plate preparation and IDBac Analysis Workflow). This search required… …analysis confirmed the genus and species assignments provided by IDBac (**Supplementary Table S2**)."

6. I think the focus of the manuscript is too broad. While the abstract suggests that IDBac is a platform for identifying bacteria (see comment #1), significant parts of the manuscript describe other applications, such as new compound discovery using comparative MANs. While I understand the aim is to describe the IDBac platform in its entirety, the enormous breadth of this publication is not helpful. This also causes problems for the reviewers. For example, I do not feel qualified to assess the validity and quality of the NMR data provided.

We addressed this understandable critique above. The manuscript title, abstract, and subheadings were adjusted to better reflect the IDBac's equally important functions.

I also doubt whether the ability of certain species to synthesize secondary metabolites, such as haereomegapolitanin or haereoacidicolin, always correlates with species assignment. In other words, it is unclear whether different metabolite production profiles are necessarily linked to different taxonomic assignments (i.e., species assignments). I think this is unlikely. In my opinion, this topic is a separate story that should not be the focus of the present manuscript.

To clarify, in this manuscript, we do not make the claim that secondary metabolite production always correlates to species assignment. Though it is well established in the field of natural product sciences that in some cases bacterial species *can be* delineated using secondary metabolite production. We revised language to clarify this point:

> "*Importantly, early in the pipeline IDBac MAN analysis indicated that two seemingly identical strains grown under the same cultivation conditions offered divergent specialized metabolite production, which* in this case *was an indicator for species-level differentiation. This highlights the power of metabolomics to* sometimes *act as a cost-effective indicator of phylogenetic divergence…*"

> *7. Knowledgebase contribution metadata sheet (Excel Metadata Sheet). In my opinion, the availability of metadata is crucial to the success of IDBac. I am therefore very pleased that the authors have devoted so much attention to this important aspect. However, the task of entering metadata should be automatable. Just imagine how long it would take to enter spectra from 100 isolates!*

We appreciate the reviewer's concern regarding the ease of use of our platform and are open to specific feedback from users on how to optimize this task. Currently, in our opinion there is no optimization that offers significant benefit beyond Excel's click and drag mechanism and autofill functionality. Additionally, we provide dropdown options for the selection of predictable metadata categories. Outside of these however, user management of data that is customized to an individual laboratory is an unfortunate reality and will require manual entry. We presume these data will already be recorded by the researcher in similar formats which will hopefully ease the burden of data entry.

Additionally, many of the common metadata fields such as cultivation time, temperature, and media are not typically provided in open spectral data formats. While automated production of metadata sheets is possible (e.g., based on sample barcoding software), we feel that this is outside the scope of this work.

> *8. Figure S23. Quality control of protein MS spectra for IDBac-KB depositions. I agree, Quality control (QC) is extremely important. However, I believe it should be performed by applying some objective criteria (please refer to Cuenod et al., 2021; or Lasch et al., 2025). Objective QC saves an enormous amount of time and effort while guaranteeing the comparability of data over a long time scale. After all, no single person (to rule out different – subjective - evaluation standards) will be able to visually assess the quality of data submitted to IDBac. Therefore, QC tools based on "hard" spectrum quality criteria should be implemented. This point could be mentioned and discussed in the manuscript (and perhaps objective QC will become a new workflow in the GNPS analysis environment in the future).*

We thank the reviewers for their helpful comments and we have included a beta version of an objective QC method inspired by Lasch et al. 2025 into IDBac. We cited this in the manuscript (reference 23). This has been deployed to the IDBac analysis and deposition workflows and we envision that this will significantly increase the ease of depositions for future users.

Minor Comments

i. Last sentence of the first text section in "Main" (line 89) Please provide a reference

Reference was added (reference 8).

ii. Second and third text sections in "Main": My impression is that other solutions for identifying bacteria are not presented in a particularly fair manner. I already pointed out at the beginning that I consider MicrobeNet to be a very easy-to-use, extremely comprehensive, helpful, and well-documented platform. There are also other possibilities to analyze bacterial spectra independently of the databases and software solutions offered by commercial providers: MALDI-UP, MicrobeMS. These solutions should also be mentioned.

We incorporated discussion of these platforms and databases in the manuscript text (l93-101) and in Table S1. In a previous response we also detailed the limitations of these and compared them to the functionality of IDBac. We hope to have achieved a more accurate comparison of available tools and databases. We thank the reviewer for this input and opine that our difference in opinion regarding these tools stems from Reviewer 2's desire to evaluate the tool mostly based on Function #1 (identification of single bacterial isolates).

Re: MicrobeNet. As discussed in earlier responses (and partially documented in Table S1), MicrobeNet is easy to use but is "comprehensive" only when concerning pathogenic microorganisms and maintains limited functionality (i.e., only Function 1 in the manuscript), among other limitations described.

iii. Results, line 153. Please describe exactly what consensus spectra are and how they are obtained.

We added language to the **Methods: IDBac-Analysis Workflow** to help guide the reader:

> "...intensities are normalized with the base peak set to 1.0 and averaged across replicates. Binned peaks present in fewer than 50% of replicates are removed to comprise a consensus spectrum for a given isolate."

iv. Results, line 183. “Spectra are searched”… As I understand it, spectra are not tested, but peak lists derived from spectra are compared. Please explain.

We apologize for the confusion and added language to **Methods: IDBac-Analysis Workflow** which details the comparison of peak lists (l188-189):

> “IDBac-Analysis provides putative bacterial identities to user-submitted protein MS spectra that are measured from either a small amount of cell material or extracted cell mass. Query spectra are processed according to **Methods: IDBac-Analysis Workflow** and searched against processed protein MS spectra of genetically characterized strains within the IDBac-KB using an intensity-agnostic cosine distance score.”

v. Line 337. Please check “(2)” after haereoacidicolin

There is no error. There is the appearance that the right parenthesis is bold; it is not.

vi. Line 376: Personally, I doubt that MALDI-ToF MS is optimally suited to be used for de-replication of strains. For de-replication purposes we made in my lab good experiences with vibrational spectroscopies, i.e., Raman or FTIR spectroscopy, as cost-effective and relatively easy-to-use methods. Compared to MALDI-ToF MS these methods are not that robust, but offer a better taxonomic resolution.

Re: other dereplication methods. We agree about the cost-effectiveness of Raman or FTIR spectroscopy and do not wish to pose IDBac as a replacement, rather an alternative that offers different analysis options (though it is also cost-effective, particularly when running batch samples). We are also currently working on integrating MS/MS analysis for further enhanced dereplication with existing ESI based databases, but this is currently beyond the scope of this report.

Re: MALDI to dereplicate isolates. We highlight the reference cited in line 385, which was our study (discussed below). Importantly, in the field of environmental microbiology (among many other non-clinical fields), there is a pressing need to compare and/or taxonomically dereplicate large numbers of unknown isolates. Several other publications employed MALDI to effectively dereplicate bacteria, many of which are referenced in the manuscript (the SpeDE package is just one example, reference 40). Just two brief examples from our own lab are provided below.

The Murphy lab (co-corresponding author) manages a bacterial natural products drug discovery program that relies on building a library of non-redundant bacteria. The original IDBac, a downloadable software package (minus most of the innovative functions presented in this

manuscript), *was originally created* to dereplicate unknown environmental bacterial isolates. In our previous study Costa et al, we employed MALDI MS to 1,616 environmental isolates to dereplicate and remove taxonomic *and* specialized metabolite redundancy. Analysis allowed us to create a minimally redundant bacterial library of 301 isolates that spanned an impressive 54 genera. Previously these types of libraries were created based only on colony morphology, which led to significant problems of redundancy. Also part of our paper, we reduced the size of an existing bacterial strain library based on the degree of metabolic overlap observed in natural product MS spectra of bacterial colonies (from 833 to 233 isolates, a 72.0% redundancy reduction!).

Additionally, our lab performs similar dereplication analyses in a collaboration with investigators from NASA's Jet Propulsion laboratory. They have been using IDBac to dereplicate isolates so that they could be prioritized for WGS (it was too costly to perform WGS on all of their spacecraft and 'clean room' isolates, and MALDI provided a cost-effective and high-throughput opportunity to sequence only non-redundant strains). They have since elected to deposit their strains in our database.

In summary, there is sufficient precedent in the literature and in our own experiences to show that MALDI MS is an effective tool to dereplicate environmental bacteria.

We thank the Editor and Reviewer 2 for their thoughtful consideration of our revised manuscript. We are particularly grateful to Reviewer 2 for once again taking the time to directly evaluate the IDBac platform and for providing constructive feedback based on this assessment. Reviewer comments are indented, italicized, and in blue font, while our responses are left aligned and in black font. No changes were needed to the manuscript file that was submitted previously. If you require any further material, we would be happy to provide it.

Reviewer #2 (Remarks to the Author):

Unfortunately, my attempts to test the IDBac platform using my own data have failed again. As the study's authors will probably be able to see in their server log files, I was again unable to test the IDBac identification pipeline. I followed the instructions in the video tutorials provided very carefully — with the result described.

I would therefore ask the authors to analyze the errors and update the documentation accordingly. While the authors responded in great detail and in a satisfactory manner to most of my comments and suggestions in the second round of review, my final assessment of the submitted study will be based mainly on the success and quality of the analyses of the IDBac workflows. This is primarily because the present study focuses on an online platform for data analysis, IDBac.

We thank the reviewer for taking the time to make use of the new documentation. We have inspected the server log files and identified that the reviewer *successfully ran* one "IDBac Analysis Workflow". They also attempted to run nine instances of the "IDBac Split MALDI Workflow", all of which failed. Our response will address these two events separately.

1. Successful IDBac Analysis Workflow. We determined that Reviewer 2 successfully ran the IDBac Analysis Workflow. Based on server logs, the workflow was submitted on March 11th at 4:47 PDT and completed successfully at 4:56 PDT. However, logs on the interactive interface show user accesses no later than 4:51 PDT (5 minutes before the run was complete). Therefore, we believe that the workflow was in a "RUNNING" state when the reviewer tried to access the interactive interface, leading to multiple error messages because the workflow had not yet reached the "DONE" state. We're certain that future users will encounter the same error and have therefore added a new error message to indicate this in real time (**Figure R1**).

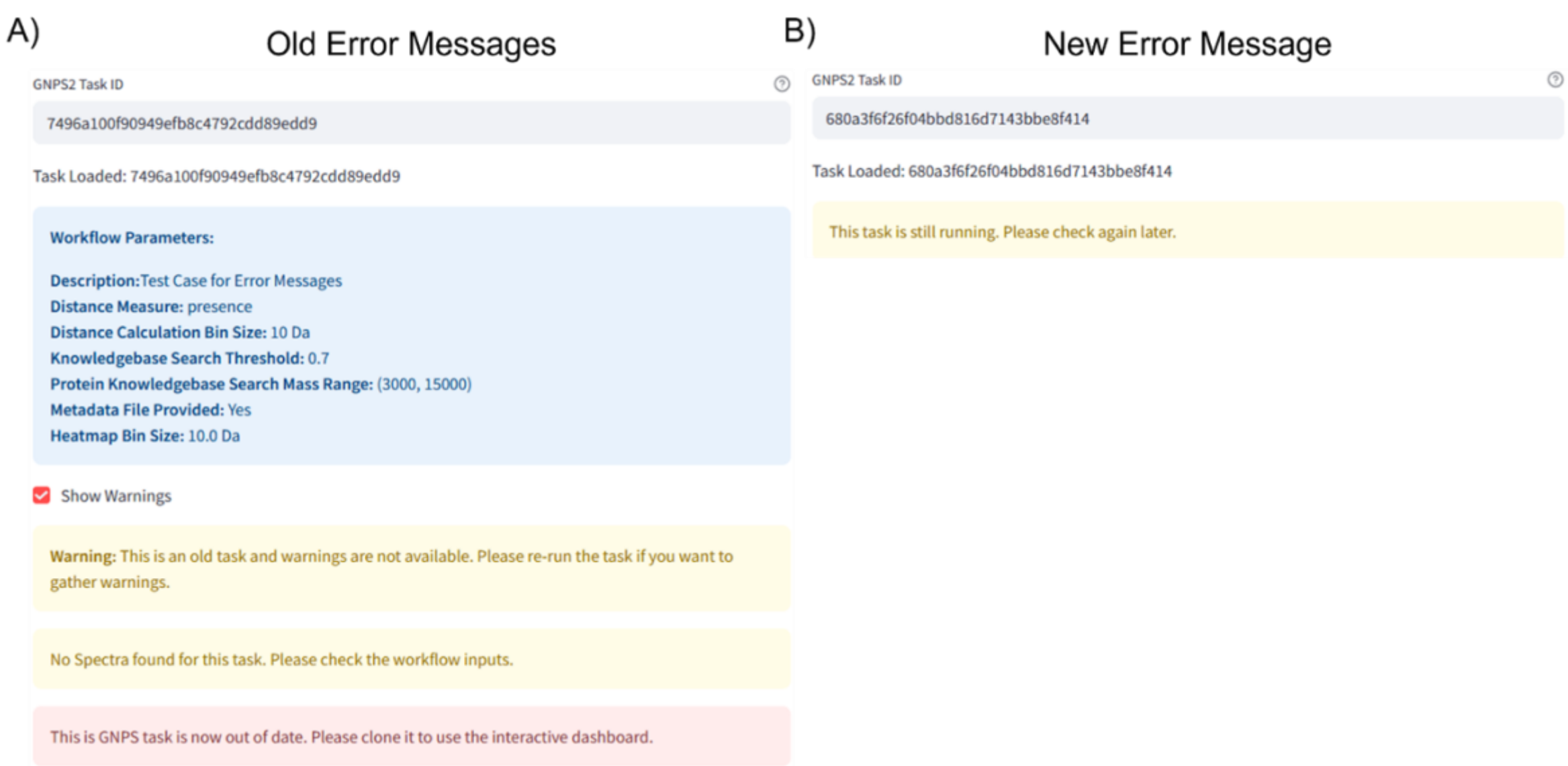

Figure R1. A) Error messages a user would have seen if the workflow was still running (prior to our error message revision). B) Revised error messages that users will see from this point forward.

Results of Reviewer 2's query: To be clear, our team did not perform these analyses for the reviewer, the following data were the result of the job Reviewer 2 submitted. Results for this task can be found at this link where the "Knowledgebase Search Summary" button can be used to view hits. The reviewer's cosine distance query matched against 107 Knowledgebase strains at their selected distance threshold of 0.65. Notably, all hits were *Brucella spp.* originally derived from the RKI collection in the IDBac-KB with the closest match being a *Brucella canis* at a cosine distance of 0.0381. A mirror plot generated on the interactive platform of this high-confidence match is shown in **Figure R2**. Interactive analysis for this task can be accessed by clicking the "Downstream Interactive IDBac Analysis" button found on the GNPS2 page or directly accessed via this URL.

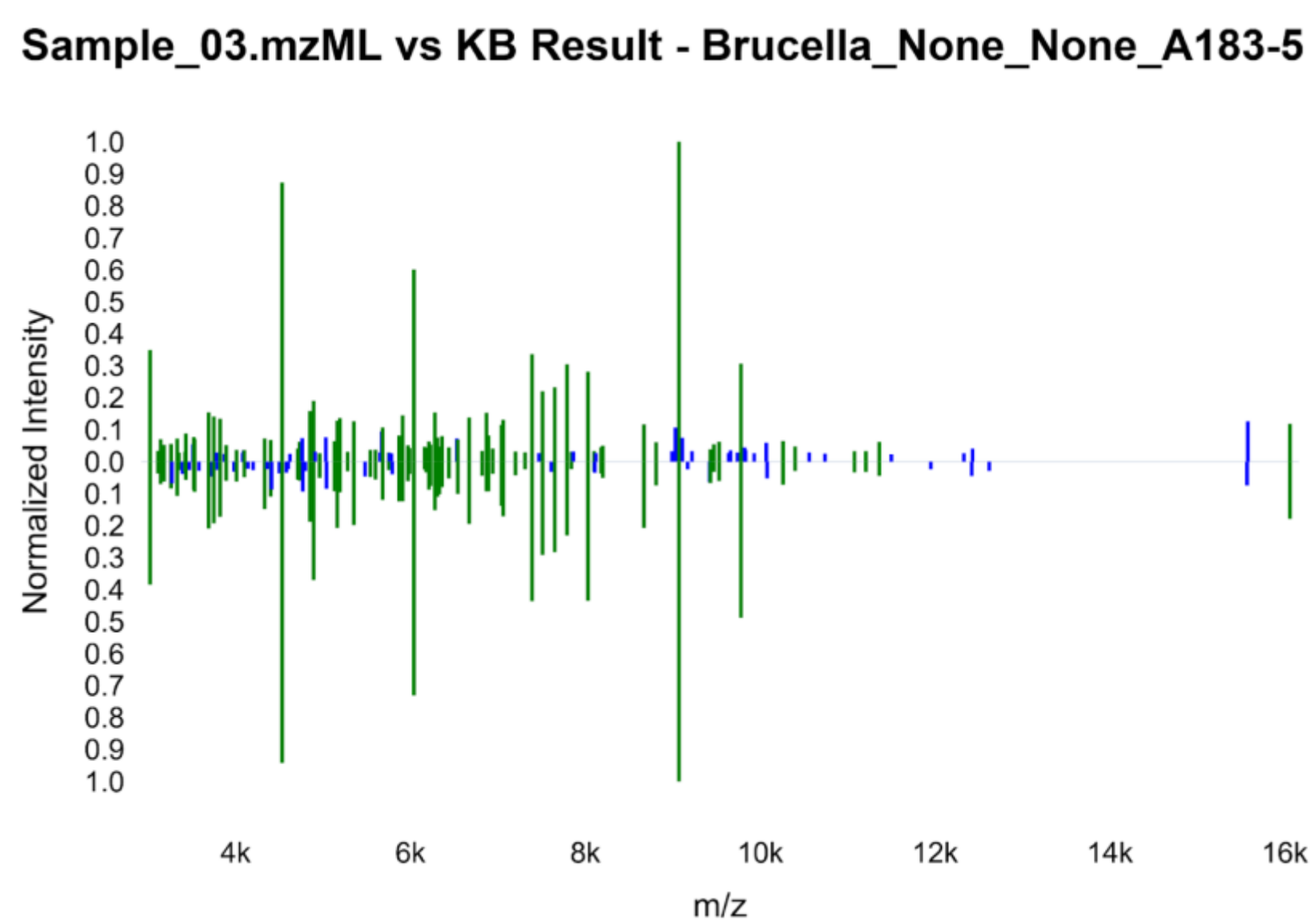

Figure R2. Mirror match of Reviewer 2's query spectra against a *Brucella canis* RKI spectrum in the IDBac knowledgebase demonstrating highly similar proteomic profiles. The calculated cosine distance was 0.0381. Matched peaks are highlighted in green, unmatched peaks are shown in blue.

One additional note about Reviewer 2's dataset: When only one strain is submitted for analysis, some IDBac features will not be fully functional (this is by design). Therefore when analyzing Reviewer 2 results at the links above, here are our comments on the additional IDBac Analysis features on the leftmost tab (we list these just in case the Editor/Reviewer 2 are curious and attempt to explore these):

Fully Functional features:

- "Protein Dendrogram" tab
- "Plot Spectra" tab

Features with reduced Functionality:

- "Protein Heatmap" tab:
  - What results look like: Heatmap is functional but the attached dendrogram is disabled.
  - Warning Message: "*There are not enough spectra to create a dendrogram. Please check the number of input spectra*."
  - Explanation: The protein heatmap requires at least 2 query strains to show a dendrogram alongside the heatmap. This functionality is therefore intentionally disabled.

Disabled features:

- "Dimensionality Reduction" tab:
  - Warning Message: "*Number of components must be less than the number of selected spectra for PCA.*"
  - Explanation: This workflow does not have enough query strains to perform dimensionality reduction analyses.

- "Metabolite Association Network" tab:
  - Warning Message: "*Small molecule analysis requires a metadata file. Please upload a metadata file first*."
  - Explanation: No small molecule data was provided with the workflow.

- "Deposition QC" tab:
  - Warning Message: "*Unable to access the data for this task. Please check the Task ID and try again*."
  - Explanation: This is not an IDBac Deposition Workflow, therefore this page is not active.

2. IDBac Split MALDI Workflow. In our evaluation of Reviewer 2's failed Split MALDI Workflow tasks, we identified two underlying problems. First, their uploaded plate map file did not follow the provided template, which is found in the IDBac documentation under Step 4 "Download a MALDI-TOF MS plate map template here" (see **Figure R3**; also found at 0:33 in the associated Step 4 video that provides rationale and guidance on how to run the Split MALDI Workflow).

In the reviewer's provided plate maps (which we presume is their own Excel file), the row labels (A-P) and column labels (1-24) were missing. These are critical since target plate coordinates are used for assigning isolate IDs to replicate scans, enabling the creation of consensus.

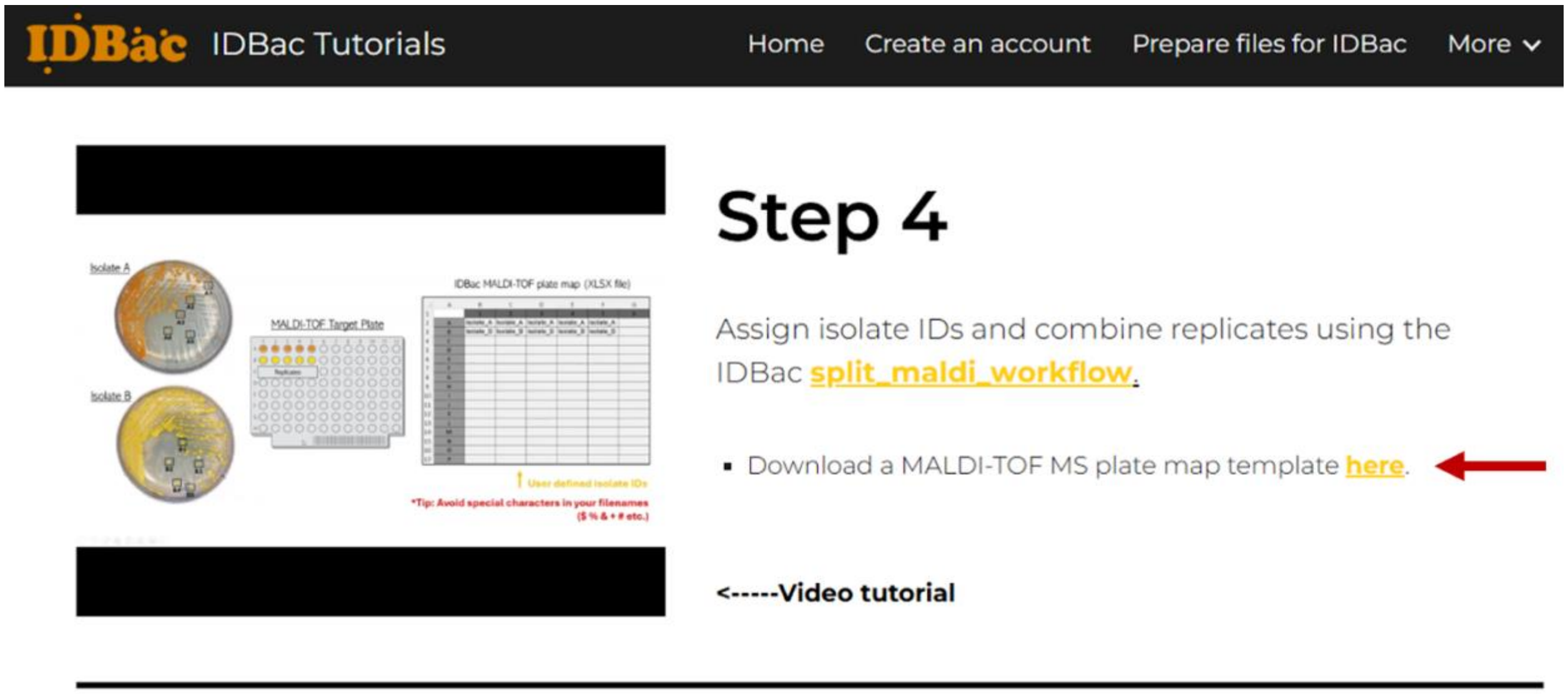

Figure R3. Screenshot of "Step 4" instructions and video.

Second, the user-provided mzML files contained data for 4 positions on a MALDI target plate while the submitted plate map accounted for only one. For convenience, we listed the well numbers for Reviewer 2 samples below:

- Sample_01.mzML: Wells H3, H4
- Sample_03.mzML: Wells H7, H8

Corrected plate maps for these samples (assuming both scans are derived from the same isolate) are provided below in **Table R1** and **R2**.

To ensure future users do not commit the same error again, we have introduced a new check within the workflow for numerical column headers and alphabetic row labels. An example of the new error message is shown for this task under "Workflow Logs" at the following link. Additionally, a 'Help' page was added to the tutorials website noting how to access these error messages. The location of new error messages is shown in **Figure R4**.

	3	4
H	Isolate_1	Isolate_1

Table R1. Corrected plate maps for Sample_01.mzML.

	7	8
H	Isolate_1	Isolate_1

Table R2. A corrected plate map for Sample_03.mzML.

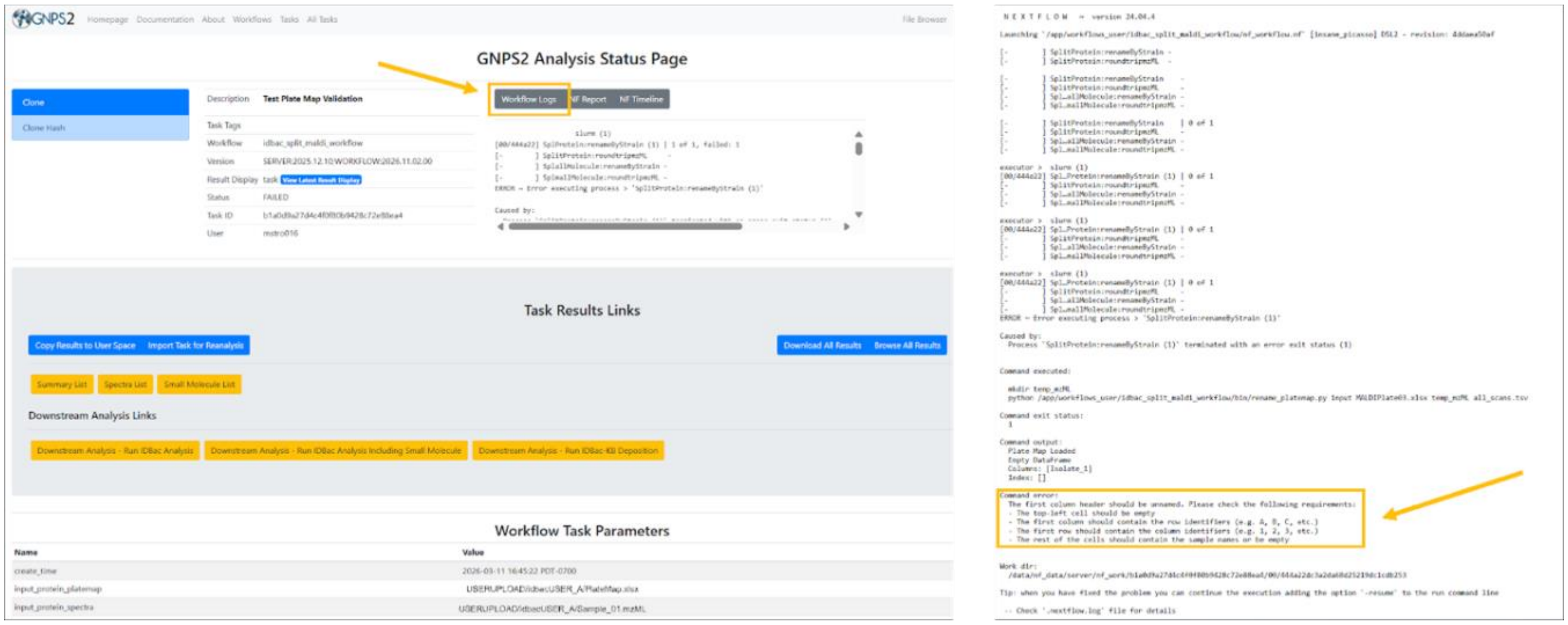

Figure R4: Location of new error messages for failed Split MALDI Workflows.

In case the Editor/Reviewer 2 would like to review results of these analyses, we reran the Split MALDI Workflow using the Excel template provided in the workflow. Please find these completed tasks using the "Corrected" platemaps at the following URLs:

- Sample_01.mzML:
    - Failed Task URL

- Failed Task Input Files:
    - MALDIPlate03.xlsx
    - Sample_01.mzML
- Corrected Task URL

- Sample_02.mzML:
    - Failed Task URL

- Failed Task Input Files:
    - MALDI Plate map 01.xlsx
    - Sample_03.mzML
- Corrected Task URL

Although reviewers #1 and #3 carefully evaluated the manuscript, they obviously did not attempt to test the platform itself. It is my view that one of the reviewers should test the IDBac platform before the study is published in the prestigious journal Nature Communications. So this task now falls to me.

We hope that we have correctly identified the errors that Reviewer 2 encountered during their evaluation. We had yet to encounter these errors with the current IDBac userbase, so this experience showed us a few additional possible user experiences. Although no major changes to the documentation and video tutorials were necessary, we added several error messages that may help

prevent user missteps in real time. We will continue to modify our workflow and improve usability as our userbase expands. But we hope that this example of a user-designer feedback loop is indicative of how we plan to maintain the IDBac platform moving forward.

Once again, we thank the reviewer for their thoughtful and comprehensive evaluation of our manuscript and platform. We feel that Reviewer 2's comments exemplify the rigorous and constructive spirit of peer review, and we are encouraged to see such a careful engagement with the work.

Reviewer 2

NCOMMS-25-72074-T, "IDBac: an open-access web platform to identify bacteria and analyze relationships in culture collections using MALDI-TOF mass spectrometry" by Krull, N.K. et al. Thank you very much for the detailed feedback. After reviewing the provided explanations and notes, I successfully tested the IDBac pipeline with my own data. The authors will be able to see from the server log files that I used MALDI-ToF mass spectra from Paraburkholderia caldeonica and a published MALDI-ToF MS dataset containing spectra of highly pathogenic bacteria for the tests. The latter dataset is available at the following link: https://wiki.microbe-ms.com/uploads/ring-trial-RKI-spectra.zip. The corresponding publication is from 2014: https://doi.org/10.1128/JCM.00813-15.

The dataset containing spectra of highly pathogenic bacteria was selected because, while revising their manuscript, the authors had in the meantime integrated mass spectra from the RKI ZENODO Database 4.2 into IDBac. This ensured that the species included in the test dataset are represented in the IDBac database, which I considered a first prerequisite for evaluating the identification methods in IDBac. Second, the fact that the identical RKI spectra are also fully available to both tools, MicrobeNet and MicrobeMS allowed conducting comparative analyses of identification accuracy between IDBac and the latter two tools.

The selection of MALDI test data for Paraburkholderia caldeonica was based on a similar rationale. The aim was to ensure that spectra for this bacterial species were part of the IDBac database. Paraburkholderia caldeonica was studied earlier in a paper by the Linington group I knew before this review (see https://doi.org/10.1128/AEM.00354-20); some of them are co-authors of the present manuscript.

In general, it can be said that the identification of bacterial isolates at the species level worked well in my tests. Identification was successful with the MALDI test data for Paraburkholderia caldeonica but there were some difficulties when interpreting the data from the test set of highly pathogenic bacteria.

We thank the reviewer for taking the time to test our platform on validated data. We are pleased to hear that the identification results worked as expected.

Performing the analyses seemed to me to be time-consuming overall. This is not only due to the need to first convert the MALDI spectra into the mzML format using MSConvert. The metadata must also be entered through a multi-step process that also looked rather complicated to me (though I did not test it).

Re: file conversion. One advantage to working with proprietary platforms (e.g., Bruker Biotyper) is that a file conversion step is not necessary. However, proprietary systems preclude researchers from sharing data across multiple platforms. We agree that this file conversion step is not ideal, however it is a current necessity and is aligned with FAIR principles. Anyone may download our protein library and utilize it for further workflows.

Re: metadata. The only metadata entry that is required are file names and a sample plate map; otherwise, all remaining metadata entry is optional. As we addressed in a previous response, metadata entry in the environmental microbiological sciences is unlikely to become less laborious over time, however the payoff for proper curation of metadata has enabled advances such as AlphaFold (as a successful example from an adjacent field). One of the unique functions IDBac provides to users is the opportunity to compare tens to thousands of bacteria while simultaneously overlaying user selected metadata properties. Examples of this payoff are provided in the main manuscript (Fig. 4). To streamline the user experience, we provide an Excel

template for data entry accompanied by precise video instructions. We also wish to note that most researchers who study non-clinical bacteria have already recorded metadata on isolates in their collection; this is part of standard workflows for collecting environmental bacteria.

> Overall, I found IDBac to be rather unintuitive. The fact that it took me four attempts just to get the pipeline up and running does not speak well of IDBac's user-friendliness. In my opinion, it would have saved everyone time and effort if the authors had consulted individuals (microbiologists?) outside of the IDBac programming team for testing before submitting their publication. Unfortunately, I had the impression that I was the first external tester of the identification pipeline.

We agree with Reviewer 2's suggestion to consult microbiologists, however many of the contributing authors of this paper are microbiologists and chemists and deposited their own data and beta tested as an inclusion requirement for authorship. It is critical to read the documentation ahead of testing when attempting any new workflow. Without following the documentation, problems will arise.

We note that through multiple rounds of review we used this feedback to implement more guardrails to prevent further user non-compliance. Additionally, we posted incremental ~3-minute screen recordings that walk users step-by-step through file conversion and data processing within each IDBac function. These greatly improved our platform and we thank Reviewer 2 for inspiring these changes.

> During my tests, I often wondered why the analyses stopped at certain points. Clear error messages and progress indicators would have been helpful. With progress indicators or an error message, for example, I would have immediately known that the analysis of a single dataset was not yet complete after four minutes of runtime and that I simply needed to be more patient. The authors shouldn't take this as the grumbling of a disinterested reviewer—I actually do really think that a lot of potential pipeline users will give up after 10 minutes (and never come back) if things don't work right away. On the other hand, I really liked the range of features in IDBac.

As part of the changes from the prior response document, we added an error message that details the user task is still running. This was very helpful feedback.

Additionally, we'd like to highlight the result progress window that shows process names and the number of remaining tasks to be completed to the workflow (see below). Unfortunately, Nextflow, the underlying workflow manager we use currently has a tracked bug that truncates the text in our output, however, we hope to address this as soon as a fix becomes available.

Workflow Logs NF Logs NF DAG NF Report NF Timeline

```
[91/5bed5c] protein:summarizeSpectra        | 1 of 1
[87/37eb96] protein:downloadDatabase        | 1 of 1
[76/baef2c] protein:databaseSearch          | 1 of 1
[d6/5592e1] proin:downloadDatabaseSummary | 1 of 1
[98/63efc4] pron:enrichCoreDatabaseSearch | 1 of 1
[5c/5dcbae] prorichCompleteDatabaseSearch | 1 of 1
[2f/9ff84c] protein:createDendrogram (1)    | 1 of 1
Completed at: 15-Apr-2026 20:40:58
Duration    : 6m 2s
```

The IDBac standard specifications were used for the identification tests. The results obtained are generally satisfactory. However, the accuracy of identifying the highly pathogenic test set appears to be slightly lower than analyses performed by other free tools, such as MicrobeNet and MicrobeMS. See the appendices for the corresponding analyses and comparison.

In our analysis of the reviewer's data, while we agree that mixed taxonomies are present at higher distances within the results, *all #1 hits were correct*. Of course, as is the case with any public user-contributed database, results will improve as the knowledgebase grows. We anticipate publication of this manuscript will greatly contribute to public KB expansion.

GNPS2 Task ID: 6d77ff26cef54fa3b4b0f968b458f243

Filename	Taxonomy From RKI Ring Trail Study<br>PMID: 26063856	Top-1 IDBac Hit
Sample_01.mzML	*Burkholderia pseudomallei* A101-10	*Burkholderia pseudomallei*
Sample_02.mzML	*Francisella tularensis* subsp. *holarctica* Ft 32	*Francisella tularensis*
Sample_03.mzML	*Brucella canis* A183-5	*Brucella canis*
Sample_04.mzML	*Bacillus anthracis* AMES	*Bacillus anthracis*
Sample_05.mzML	*Ochrobactrum anthropi* A148-11	*Brucella anthropi**
Sample_06.mzML	*Yersinia pseudotuberculosis* type III	*Yersinia pseudotuberculosis*
Sample_07.mzML	*Burkholderia mallei* A106-3	*Burkholderia mallei*
Sample_08.mzML	*Burkholderia thailandensis* E125	*Burkholderia thailandensis*

Sample_09.mzML	*Yersinia pestis* A106-2	*Yersinia pestis*
Sample_10.mzML	*Bacillus thuringiensis* DSM350	*Bacillus thuringiensis*

* In 2020, after publication of the RKI Interlaboratory Ring Trial Study the American Society for Microbiology reclassified *Orchobatrum antropi* as *Brucella antropi* (She et al., 2020).

I can only speculate on the reasons for this discrepancy. One possibility is that using a very wide window of 10 Th for peak detection is not optimal. Furthermore, the test set is rather challenging, due to the presence of closely related species like Yersinia pestis/ Y. pseudotuberculosis, Bacillus species, or species from the Burkholderia pseudomallei group. However, these are precisely the types of situations that tools like IDBac will encounter in routine use.

Without further details about the observed discrepancy, it is difficult for us to comment further. If the reviewer is referring to the mixed taxonomy at higher distance levels in knowledgebase search results, we anticipate that for highly-related taxa, cut-off distances will vary. This limitation is covered in the manuscript: "*We note that when using cosine distance to evaluate protein MALDI MS spectral similarity, there is no universal threshold to define genus/species boundaries between taxa.*"

Additionally, we would like to highlight that the current IDBac analysis workflow allows users to select from a variety of window widths under the "Similarity Calculation Bin Size (Da)" option. However, in practice we have found that 10 Da provides balanced performance in both genus- and species-level annotation.

Unfortunately, I had difficulty determining the species identified based on the file names displayed in the IDBac score lists. Only some of the metadata from the RKI database spectra was carried over during import into IDBac. Therefore, interpreting the score lists required considerable effort. The authors should address the missing metadata issue in a future update.

It would also be desirable if the results of the analyses were presented in a clearer manner. In the default setting, for example, the score lists display all analyzed test dataset results in a single table. Although the display of results can be manually restricted to individual mzML test data, I could not find a setting that allows for a clear presentation of identification results for more than just one test spectrum, as is available in MicrobeNet or MicrobeMS.

Respectfully, the knowledgebase summary page displayed on GNPS2 provides IDBac-KB taxonomy in addition to strain names (see example in Fig. 1 below). However, we acknowledge that this display format is geared towards rapid, rather than in depth analysis and previously incorporated a "Search Results" page on analysis.idbac.org that offers modifiable columns, including "KB Genus" and "KB Species" (see Fig. 2).

Query Filename	Knowledgebase Strain Name	Knowledgebase Culture Collection	Knowledgebase Taxonomy	Distance
Search Query Fil	Search Knowledgebase Strain Name	Search Knowledgeb	Search Knowledgebase Taxonomy	Search Distance
WMMA1923.mzML	K633	Murphy Lab UIC	Micromonospora sp. (User Submitted 16S)	0.5323945796903191
WMMA1923.mzML	129A-35	Murphy Lab UIC	Micromonosporaceae;Micromonospora;Micromonospora sp.	0.6372757805431917
WMMA1923.mzML	K779	Murphy Lab UIC	Micromonospora sp. (User Submitted 16S)	0.5312272095638247
WMMA1923.mzML	K792	Murphy Lab UIC	Micromonospora sp. (User Submitted 16S)	0.5394270864983142
WMMA1923.mzML	K622	Murphy Lab UIC	Micromonospora sp. (User Submitted 16S)	0.5795005077556947
WMMA1923.mzML	139A-37	Murphy Lab UIC	Microbacteriaceae;Agrococcus;Agrococcus sp.	0.6384211346489368
WMMA1923.mzML	K787	Murphy Lab UIC	Micromonospora sp. (User Submitted 16S)	0.6095383503410817
WMMA1923.mzML	K701	Murphy Lab UIC	Micromonospora sp. (User Submitted 16S)	0.5289606446933282

Fig 1. The Knowledgebase Search Summary Page on GNPS2. A screenshot of what users can expect to see on the GNPS2 knowledgebase search result page demonstrating the inclusion of taxonomy in displayed results.

Search Results

This page shows your knowledgebase search results in table form.

☑ Add filters

Filter dataframe on

Query Filename × Distance ×

Substring or regex in Query Filename

WMMD1128

Values for Distance

0.01 0.65

0.01 0.99

	Distance	Query Filename	KB Strain Name	KB Culture Collection	KB Genus	KB Species	KB Genbank Accession	NCBI Taxid	KB Sa
52	0.6377	WMMD1128.mzML	K633	Murphy Lab UIC	Micromonospora	sp.	None	None	None
104	0.6255	WMMD1128.mzML	129A-35	Murphy Lab UIC	Micromonospora	Micromonospora sp	MK168037	1876	None
208	0.6298	WMMD1128.mzML	K779	Murphy Lab UIC	Micromonospora	sp.	None	None	None
260	0.519	WMMD1128.mzML	K792	Murphy Lab UIC	Micromonospora	sp.	None	None	None
416	0.6068	WMMD1128.mzML	K622	Murphy Lab UIC	Micromonospora	sp.	None	None	None
624	0.5544	WMMD1128.mzML	K787	Murphy Lab UIC	Micromonospora	sp.	None	None	None
676	0.6013	WMMD1128.mzML	K701	Murphy Lab UIC	Micromonospora	sp.	None	None	None

Fig 2. The "Search Results" Page on the IDBac Interactive Interface. A screenshot of the search results as shown on the interactive interface where users can readily view results for each of their query strains. Shown is a user filtering for their query strain, "WMMD1128", and filtering for distances less than

0.65. In the results, users can see the genus and species of their hits, along with GenBank accession (if available).

Distance estimation using cosine distance appears to work very well. It is an interesting way to further refine identifications based on spectroscopy and/or spectrometry, not only for bacteria. The dendrograms from the cluster analysis include reference entries from the IDBac database. While this may be helpful in certain scenarios, it would also be helpful to have the option to exclude such database spectra from these analyses and / or visualizations.

We thank the reviewer for raising this issue and agree that this functionality is useful to end users. To achieve this in IDBac, please follow these steps to remove the knowledgebase results from the dendrogram: On the "Protein Dendrogram" page, locate the "Knowledgebase Search Results" dropdown and deselect "Show All".

On the other hand, I really liked the visualization of the PCA results. The phylogenetic relationships of the species in the test dataset are adequately reflected in the score plots.

QT: Unfortunately, the results of the quality test are not comprehensible. However, since this pipeline was explicitly marked as beta, I will not address these analyses in my review. Nevertheless, improvements of the QT pipeline are definitely required.

We thank the reviewer for the feedback and we are actively working to improve this portion of the pipeline.

Comparable tools, such as the MALDI Biotyper (MBT), MicrobeNet, and MicrobeMS, allow performing identification analyses based on individual MALDI spectra, not only of consensus spectra. When evaluating identification results, it is often useful to be able to analyze replicate spectra of a sample individually. Therefore, the pipeline should offer an option to analyze single MALDI-ToF mass spectra.

While this is currently feasible using the split workflow (users can individually label each replicate on their plate map i.e IsolateA_1, IsolateA_2 etc.), we recognize the utility of visualizing consensus spectra *and* replicates within the same analysis run. We are interested in supporting this feature in future versions of IDBac, and it may be achievable with significant changes to the underlying data representation.

As a follow-up to the topic of dereplication: We also gained experience in this area in our laboratory. In various projects, we faced the challenge of how to select phenotypically highly similar isolates from a large number of bacterial samples for subsequent whole-genome sequencing (WGS) analyses. In short, our experience indicates that the taxonomic resolution of MALDI-ToF MS is inferior to that of spectroscopic methods, such as Raman and infrared spectroscopy. With comparable effort in data acquisition and analysis, these techniques enable significantly better differentiation between closely related yet distinct strains than MALDI-ToF MS does. One application scenario for the IR Biotyper, developed by Bruker Daltonics, consequently involves dereplication of bacterial isolates in outbreak investigations. Furthermore, some years ago, RiverDiagnostics, a Raman company in the Netherlands, introduced a corresponding Raman solution for the dereplication in clinical settings.

However, I'm not denying that MALDI ToF MS is suitable for dereplication tasks. However, if I had to choose, I would always use FTIR or Raman spectroscopy for these tasks.

Related literature:

https://doi.org/10.1016/j.ijfoodmicro.2025.111550
https://doi.org/10.3389/fmicb.2026.1735218

We thank the reviewer for highlighting the capabilities of FTIR and Raman spectroscopy. We appreciate the provided references and are in agreement that these vibrational spectroscopic methods can offer excellent sub-species taxonomic resolution.

However, we maintain that MALDI-TOF MS remains a robust and inherently valuable tool for dereplication, particularly for the scale and context of our environmental study. While it is true that all phenotypic methods (including MALDI-TOF MS) require standardized culture conditions to ensure reproducibility, the whole-cell biochemical profiling of FTIR and Raman systems is liable to decrease sensitivity under minor methodological variations. In contrast, MALDI-TOF MS targets highly conserved ribosomal proteins providing a more robust "first-pass" filter (Wenning et al., 2014).

Summary: After thorough consideration, it is my estimation that the publication of the manuscript is advisable in its present form. It is my hope that the authors will take my suggestions into account and consider my recommendations when updating IDBac in the future. The initiative to provide free, vendor-independent, and publicly accessible pipelines for MALDI-ToF MS analyses in microbiology, along with the related resources, is very much welcomed. Notwithstanding the presence of certain outstanding tasks and deficiencies that require attention, it is my conviction that the present manuscript merits publication in its current state.

Again, we thank the Reviewer 2 for taking the time to carefully review our manuscript and the platform itself. Their feedback and ideas have allowed us to make improvements to both.
